# Development of Novel Honey- and Oat-Based Cocoa Beverages—A Comprehensive Analysis of the Impact of Drying Temperature and Mixture Composition on Physical, Chemical and Sensory Properties

**DOI:** 10.3390/molecules29194665

**Published:** 2024-09-30

**Authors:** Kristina Tušek, Maja Benković

**Affiliations:** 1 Health Centre Krapina-Zagorje County, Mirka Crkvenca 1, 49000 Krapina, Croatia; tusek.kristina@gmail.com; 2 Faculty of Food Technology and Biotechnology, University of Zagreb, Pierottijeva 6, 10000 Zagreb, Croatia

**Keywords:** cocoa powder, antioxidant capacity, polyphenols, oat, honey

## Abstract

This research aimed to assess the influence of drying temperature (50, 60 and 70 °C), honey/oat flour ratio (60:40, 50:50 and 40:60) and cocoa contents (5, 6.25 and 7.5 g/100 g) on the physical (color, moisture content, bulk density, flowability (Hausner ratio, Carr index), dispersibility, solubility, and particle size), chemical (total dissolved solids, conductivity, pH, amount of sugar, color, total polyphenolic content, and antioxidant activity), and sensory properties (powder appearance, color, odor; and beverage appearance, color, odor, sweetness, bitterness, taste, texture) of a newly developed cocoa powder mixture in which honey was used as a sweetener and oat flour as a filler. Also, a further aim of this study was to optimize the composition of the mixture based on chemical, physical and sensory properties. Based on the optimization results, the highest total polyphenolic content and antioxidant activity were achieved at 70 °C with a honey/oat ratio of 50% and a cocoa content of 7.5 g. Drying temperature has a significant effect on powder odor and beverage odor, as well as on beverage bitterness, while the honey/oat flour ratio has a significant effect on color, with primarily values L* and a*. The cocoa contents mostly affect total polyphenolic content and antioxidant activity.

## 1. Introduction

Research consistently underscores the pivotal role of a proper and balanced diet in promoting better health outcomes and longevity. Indeed, eating habits are profoundly influenced by various factors, including community norms, family traditions, personal preferences, and the accessibility of food options. This intricate interplay of socio-cultural, economic, and individual factors shapes dietary behaviors and patterns across populations. Recognizing these influences is crucial for designing effective strategies to encourage and facilitate healthier eating habits on both individual and societal levels. One of the strategies is the development of novel foods, in which the highly processed industrial ingredients are replaced by minimally processed, locally available ingredients. This strategy not only produces functional foods with better nutritional properties, but also aids the ever-growing fight to prevent climate change, since local, minimally processed plant ingredients are also considered to have a low carbon footprint [1].

According to literature data, the same environmental trend is also seen in the cocoa production and processing industry. Besides the already widely present alliances for sustainable cocoa production (e.g., Rainforest Alliance, Cocoa Alliance and similar), the development of functional, novel cocoa beverages containing sustainable, locally based ingredients with a low carbon footprint has also been a focus of researchers around the world [2,3,4]. Beyond its sensory appeal (flavor, aroma, and texture), cocoa has attracted considerable attention for its potential health benefits owing to its rich profile of bioactive compounds. Cocoa is a well-known source of polyphenols, especially flavanols, as well as methylxanthines, phytosterols, and dietary fibers [5]. It is also a rich source of lipids (24–10%), proteins (20–15%), carbohydrates (15%), and micronutrients, both minerals (P, Ca, K, Na, Mg, Zn, and Cu) and vitamins (A, B, E) [6]. Being rich in both macro- and micronutrients, cocoa is linked to a variety of health-promoting activities, including antioxidant, anticarcinogenic, antidiabetic, anti-inflammatory, anti-obesity, and anti-allergenic effects, which may contribute to the overall well-being [5].

Similar to cocoa, honey is also rich in antioxidant molecules, including phenolic compounds, such as flavonoids and phenolic acids [7]. Several in vitro and in vivo studies have shown that honey possesses antimicrobial, antiviral, antifungal, anticancer, and antidiabetic properties. Furthermore, honey has been proven to have protective effects on the cardiovascular, nervous, respiratory, and gastrointestinal systems and also has a protective effect in physiological conditions characterized by high levels of free radicals [8,9,10].

Oat provides important amounts of carbohydrates, mainly in the form of starch, dietary soluble fiber, lipids, proteins and several B vitamins [11]. The consumption of oat products has been associated with a reduction in serum cholesterol, and thereby with a reduction in the risk of cardiovascular diseases, as well as diabetes and gastrointestinal disorders [12]. Furthermore, oats are the only cereal that contains saponins, specifically unique steroidal glycosides known as avenacosides A and B, which have been proven to have anticancer activity through various complex mechanisms [13].

The process of honey drying remains a challenging task for both researchers and the industry. Over the past decade, many studies have addressed this issue; however, a method for producing natural honey powder without added carriers has yet to be developed [14]. The primary challenge in honey drying is the “stickiness problem,” which is associated with the low glass transition temperature of low-molecular-weight sugars (such as sucrose, maltose, glucose, and fructose, the main components of honey) [15]. During conventional drying at elevated temperatures, even with a low water content, these sugars can result in a product with a rubbery consistency, leading to highly hygroscopic powders prone to stickiness and flow issues [14]. So far, the primary method to address this problem has been the addition of high-molecular-weight carriers (such as maltodextrin and gum Arabic), which are characterized by high glass transition temperature values [16,17]. This addition raises the glass transition temperature value of the material above the drying temperature, allowing the material to achieve a glassy state and produce a powdered product. Depending on the type of carrier, the minimum addition is about 35–50% of the feed solids [15], which means that a significant amount of carrier material is incorporated into the final product, which can affect its properties. Another theoretically possible method is to reduce the drying temperature below the glass transition temperature, which greatly increases the duration of the process and reduces its efficiency and economic profitability [14]. Also, it is important to note that the harmful effect of heating on honey is proportional to the temperature and duration of the applied heat; the higher the temperature and the longer the duration, the greater the deficiency in honey quality and its biological properties [18].

Unlike honey, oat proteins are thermostable. Oat globulin denatures at approximately 114 °C, and oats possess excellent hydration and emulsifying properties. The stability of the flavor of oat products depends on their lipid composition and resistance to oxidation [19]. Therefore, deactivating lipase and lipoxygenase through steaming, baking, or frying is crucial to prevent the unpleasant taste of oat flour [20]. Although oats contain a high proportion of lipids, natural antioxidants like vitamin E help maintain their stability and prevent oxidation. However, improper storage can lead to lipase activation, causing rancidity in oats and their final products, thus shortening their shelf life [20]. Additionally, oats are rich in starch and β-glucan [21], which provide gelling and thickening properties. These characteristics allow oats to be used as a natural, functional thickener or food gel that does not require extensive pre-processing, unlike many commonly used thickeners in cocoa beverages.

Following the above-mentioned facts, this research aimed to assess the influence of drying temperature (50, 60 and 70 °C), honey/oat flour ratio (60:40, 50:50 and 40:60) and the cocoa contents (5, 6.25 and 7.5 g/ 100 g) on the physical, chemical and sensory properties of a newly developed cocoa powder mixture in which honey was used as a sweetener and oat flour as a filler. Also, a further aim of this study was to optimize the composition of the mixture based on chemical, physical and sensory properties.

## 2. Results and Discussion

In this work, the effect of the mixture composition and drying temperature on the physical, chemical and sensory properties of newly developed, honey- and oat-based cocoa powder mixtures was analyzed. The results are presented for each group of properties separately.

### 2.1. Physical Properties of the Cocoa Powder Mixtures

The physical properties, including color, moisture content, bulk density, flowability (Hausner ratio, Carr index), dispersibility, solubility and particle size, were analyzed. The results are presented in Table 1.

#### 2.1.1. Color Measurement

The color of cocoa powders is known to be an important indicator of the processing conditions of the cocoa powders, as well as an important property which makes the cocoa powder appealing to consumers. As shown in Table 2, the L*-color coordinate values for samples dried at T = 50 °C ranged from 31.32 ± 0.01 (sample 50-2) to 56.55 ± 0.25 (sample 50-3). For samples dried at T = 60 °C, the L*-color coordinate values ranged from 42.67 ± 0.32 (sample 60-6) to 54.74 ± 0.131 (sample 60-9), while for the samples dried at T = 70 °C, the L* ranged from 38.3 ± 0.02 (sample 70-5) to 48.42 ± 0.01 (sample 70-4). According to the measurements, the samples containing less cocoa and with a honey/oat flour ratio of 40:60 are lighter. The results obtained also indicate that by increasing the drying temperature, the samples become lighter. For samples dried at T = 50 °C, the a* value ranged from 13.64 ± 0.03 (sample 50-3) to 16.48 ± 0.01 (sample 50-1), and for samples dried at T = 60 °C, the a*-color coordinate values ranged from 13.87 ± 0.01 (sample 60-8) to 17.83 ± 0.02 (sample 60-7), while for the samples dried at T = 70 °C, the a* ranged from 13.97 ± 0.01 (sample 70-4) to 16.51 ± 0.01 (sample 70-5). It is obvious that all a* color coordinate values are positive, which means that the color of the samples is in the red domain. For all samples, the b* values are positive, meaning that the color of the samples is in the yellow domain and the values range as follows: for samples dried at T = 50 °C, from 14.69 ± 0.01 (sample 50-2) to 20.11 ± 0.02 (sample 50-4); for samples dried at T = 60 °C, from 17.71 ± 0.01 (sample 60-3) to 19.36 ± 0.01 (sample 60-2); and for samples dried at 70 °C, from 16.71 ± 0.01 (sample 70-8) to 18.39 ± 0.01 (sample 70-2). The chroma value represents color saturation, and the values for samples dried at T = 50 °C ranged from 21.38 ± 0.02 (sample 50-2) to 25.31 ± 0.02 (sample 50-1); for samples dried at T = 60 °C, it ranged from 22.71 ± 0.01 (sample 60-3) to 25.56 ± 0.035 (sample 60-7); and for samples dried at 70 °C, it ranged from 22.21 ± 0.01 (sample 70-6) to 26.18 ± 1.72 (sample 70-5). The hue value represents the tone of the color and according to the measurements, the values for samples dried at T = 50 °C ranged from 43.30 ± 0.001 (sample 50-2) to 53.97 ± 0.01 (sample 50-3), for samples dried at T = 60 °C, these ranged from 45.76 ± 0.02 (sample 60-7) to 53.36 ± 0.030 (sample 60-4), and for samples dried at 70 °C, these ranged from 48.78 ± 0.02 (sample 70-8) to 51.06 ± 0.02 (sample 70-4). The cocoa component is distinctive in providing both flavor and color to final products. In this case, alkalized cocoa, which is usually darker [22], provided an intensive darker color which was especially pronounced in samples with high cocoa contents. Oat, on the other hand, diminished the darkness of the samples, which makes the determination of the ratio of oat addition one of the most important parts of mixture design when it comes to color.

#### 2.1.2. Moisture Content and Water Activity

In the production of powders, it is important that the moisture content is adjusted to maintain the quality of the powder due to the fact that the cost of the milling process generally depends on the moisture content of the samples. Water makes materials softer and acts as a plasticizer; therefore, materials with a lower moisture content break and crumble more easily [23].

The results of the moisture content measurement are shown in Table 2. The percentage of moisture ranged from 6.95 ± 0.06 (sample 50-3) to 11.33 ± 0.102 (sample 50-6) for samples dried at T = 50 °C, from 4.45 ± 0.013 (sample 60-9) to 9.64 ± 0.029 (sample 60-3) for samples dried at T = 60 °C, and from 6.82 ± 0.004 (sample 70-7) to 9.75 ± 0.22 (sample 70-8) for samples dried at T = 70 °C, showing that the lowest percentage of moisture was measured for samples dried at T = 60 °C and not, as expected, for samples dried at T = 70 °C. As far as the cocoa content and honey/oat flour is concerned, no clear influence on the moisture content of the produced powders was observed.

Hii et al. [24], in their work, examined the effect of drying of the cocoa beans in a thin layer using natural sunlight and hot air inside a ventilated oven at air temperatures of 60 °C, 70 °C and 80 °C. Drying is usually stopped when the moisture content of the dry grain reaches 7.5%. Drying was carried out for 8 h a day, and the cocoa beans were left to temper at room temperature overnight. Tempering is a common routine in the drying of cocoa beans and the purpose is to redistribute the internal moisture towards the outer layer of the beans after each drying cycle. The moisture content was determined by weighing the grains before and after drying. Moisture percentages of 7.26%, 6.72%, 6.09% and 3.74% were determined for drying in the sun and in the oven at 60 °C, 70 °C and 80 °C. Furthermore, the effect of cocoa powder and steaming time on the characteristics of a cocoa beverage powder was studied by Hardiyanto et al. [25]. Two types of cocoa powder were used, a cocoa powder with a fat content of 11% and one with a content of 27%. The use of steam and the drying process changed the initial moisture content of the cocoa powder. The results showed that regardless of the type of cocoa powder, the moisture content increased with the steaming time. Compared to the moisture content of raw materials, the moisture content of instant cocoa powder was lower. This was due to the use of a drying process after steaming which led to the evaporation of water.

Water activity (aw) is a measure of the availability of water in a substance for microbial growth and chemical reactions. It is an indicator of microbial growth, enzyme activity, preservation and food quality [26]. As shown in Table 2, water activity values for samples dried at T = 50 °C ranged from 0.352 ± 0.005 (sample 50-4) to 0.51 ± 0.004 (sample 50-1), for samples dried at T = 60 °C, the values ranged from 0.234 ± 0.003 (sample 60-9) to 0.467 ± 0.007 (sample 60-3), while for the samples dried at T = 70 °C, the values ranged from 0.348 ± 0.02 (sample 70-7) to 0.487 ± 0.001 (sample 70-8), showing that the lowest value of aw was measured for samples dried at T = 60 °C, and the same was observed for the moisture content. However, as the cocoa content and honey/oat flour ratio are concerned, no clear influence was found. Lower water activity levels can inhibit the growth of microorganisms, such as bacteria, yeast, and molds, and reduce the rate of chemical reactions, thereby extending the shelf life of food products.

#### 2.1.3. Bulk Density

The bulk density of powders is the ratio of mass to volume that the powder occupies or the mass of powder that can be accommodated in a certain volume, with the volume including the volume of air between the particles and the volume of the particles themselves [27]. Therefore, the measurement of bulk density is of great importance in industry for adjusting storage, processing, packaging and distribution conditions. It is used as part of the specifications for a certain final product obtained by milling or drying [28]. Table 2 shows the values of bulk density, Hausner ratio and Carr index. The values of bulk density ranged from 627.96 ± 31.39 kgm^−^^3^ (sample 50-3) to 720.55 ± 36.03 kgm^−^^3^ (sample 50-2) for samples dried at T = 50 °C, from 675.73 ± 34.24 kgm^−^^3^ (sample 60-9) to 724.09 ± 36.20 kgm^−^^3^ (sample 60-1) for samples dried at T = 60 °C, and from 716.63 ± 35.83 kgm^−^^3^ (sample 70-3) to 769.75 ± 38.49 kgm^−^^3^ (sample 70-4) for samples dried at T = 70 °C, showing the highest values for samples dried at T = 70 °C with a honey/oat flour ratio of 40:60. It was also visible that samples dried at higher temperatures also had higher bulk density values. For cocoa content, no clear influence on the bulk density was observed.

Regarding the Hausner ratio, the values for samples dried at T = 50 °C ranged from 1.14 ± 0.057 (sample 50-1) to 1.88 ± 0.059 (sample 50-2), for samples dried at T = 60 °C, these ranged from 1.17 ± 0.058 (samples 60-3 and 60-7) to 1.26 ± 0.063 (samples 60-8 and 60-9), and for samples dried at T = 70 °C, these ranged from 1.14 ± 0.057 (sample 70-9) to 1.35 ± 0.067 (sample 70-5). A Hausner index greater than 1.4 indicates poor flowability, while a value lower than 1.25 suggests good flowability of the powder mixtures [28]. These results showed that the prepared powder mixture should not cause problems during industrial transport and storage.

The calculated values of Carr index ranged from 14.45 ± 0.72 (sample 50-1) to 24.09 ± 1.20 (sample 50-3) for samples dried at T = 50 °C, from 16.92 ± 0.846 (sample 60-7) to 26.56 ± 1.328 (sample 60-9) for samples dried at T = 60 °C, and from 12.31 ± 0.615 (sample 70-2) to 22.69 ± 1.134 (sample 70-1) for samples dried at T = 70 °C. A Carr index < 15 indicates very good flowability, and one of 15-20 indicates good flowability, while values greater than 25 are considered an indicator of poor flowability [28], according to which sample 60-9 (ratio honey/oat flour of 50:50, cocoa 6.25 g) has the poorest flowability, but the other prepared powder mixtures are acceptable both for industrial transport and storage as well as for consumers.

In contrast to the current study, where bulk density values ranged from a minimum of 627.96 ± 31.39 kg/m^3^ (sample 50-3) to a maximum of 769.75 ± 38.49 kg/m^3^ (sample 70-4), Belščak-Cvitanović et al. [29] reported slightly higher bulk density values (862.20 kg/m^3^) for their novel cocoa powder beverages. This difference can be attributed to the addition of sugar in their formulation. The lower bulk density observed in our study is likely due to the absence of crystalline sugar and the use of a combined grinding process after drying, as opposed to the dry mixing method employed in the aforementioned research.

#### 2.1.4. Reconstitution Properties

Food powder dispersibility, in this case, cocoa powder, has a direct impact on the consumer’s perception of the overall quality of the product; therefore, dispersibility control is essential to achieve high-quality powdered food products. This is especially important for cocoa powders since it is known that cocoa powder is difficult to dissolve in water due to the hydrophobic nature of cell walls and the presence of fat [30]. As shown in Table 2, the values of dispersibility ranged from 5.44 ± 0.51 s (sample 50-7) to 16.436 ± 1.19 s (sample 50-8) for samples dried at T = 50 °C, from 10.703 ± 0.88 s (sample 60-7) to 32.113 ± 4.96 (sample 60-8) for samples dried at T = 60 °C, and from 13.14 ± 3.01 s (sample 70-7) to 33.71 ± 5.07 (sample 70-5). The wettability values ranged from 14.27 ± 2.57 s (sample 50-6) to 81.74 ± 7.66 s (sample 50-3) for samples dried at T = 50 °C, from 30.175 ± 5.64 s (sample 60-7) to 261.08 ± 12.75 (sample 60-1) for samples dried at T = 60 °C, and from 11.77 ± 16.06 s (sample 70-4) to 116.87 ± 20.42 (sample 70-9). Generally speaking, better dispersibility and wettability were obtained for samples dried at T = 50 °C with a lower content of cocoa, while significant changes in dispersibility and wettability with different proportions of honey and oats flour were not determined.

#### 2.1.5. Particle Size Distribution

Particle size is one of the most important properties that affect powder behavior during handling, transportation and storage [31]. The results of particle size distribution were determined using the laser diffraction method with dry dispersion of the sample and are shown in Table 2. The values for d (0.1) (µm) ranged from 26.433 ± 1.097 µm (sample 50-3) to 174.07 ± 5.662 µm (sample 50-5) for samples dried at T = 50 °C, from 10.013 ± 0.816 µm (sample 60-8) to 183.507 ± 9.175 µm (sample 60-6) for samples dried at T = 60 °C, and from 49.384 ± 3.063 µm (sample 70-4) to 922.785 ± 61.507 µm (sample 70-9) for samples dried at T = 70 °C. For parameter d (0.5) (µm), the values for samples dried at T = 50 °C ranged from 219.556 ± 6.35 µm (sample 50-3) to 470.112 ± 95.97 µm (sample 50-2), for samples dried at T = 60 °C d (0.5), the values ranged from 185.619 ± 7.726 µm (sample 60-8) to 792.727 ± 39.636 µm (sample 60-6), and for samples dried at T = 70 °C the d (0.5), the values ranged from 274.811 ± 17.377 µm (sample 70-4) to 1279.54 ± 24.301 µm (sample 70-9). For parameter d (0.9) (µm), the values for samples dried at T = 50 °C ranged from 572.376 ± 22.10 µm (sample 50-3) to 906.71 ± 45.407 µm (sample 50-8), for samples dried at T = 60 °C, the d (0.5) values ranged from 514.527 ± 27.726 µm (sample 60-4) to 1454.755 ± 72.74 µm (sample 60-6), and for samples dried at T = 70 °C, the d (0.9) values ranged from 801.867 ± 35.059 µm (sample 70-7) to 1653.99 ± 34.192 µm (sample 70-9). The values for D [3,2] (µm) ranged from 64.797± 1.73 µm (sample 50-3) to 319.312 ± 9.099 µm (sample 50-5) for samples dried at T = 50 °C, from 47.478 ± 1.542 µm (sample 60-8) to 362.576 ± 18.129 µm (sample 60-6) for samples dried at T = 60 °C, and from 95.862 ± 4.716 µm (sample 70-4) to 1091.322 ± 98.58 µm (sample 70-9) for samples dried at T = 70 °C. For parameter span (µm), the values for samples dried at T = 50 °C ranged from 1.528 ± 0.021 µm (sample 50-5) to 2.487 ± 2.48 µm (sample 50-3), for samples dried at T = 60 °C, the span values ranged from 1.604 ± 0.592 µm (sample 60-6) to 2.687± 0.091 µm (sample 60-8), and for samples dried at T = 70 °C, the span values ranged from 0.571 ± 0.069 µm (sample 70-9) to 2.726 ± 0.078 µm (sample 70-5).

According to the values, span is inversely proportional to all other particle size distribution parameters (d (0.1), d (0.5) (µm), d (0.9), D [3,2] (µm)); the narrower the distribution, the smaller the span becomes [32]. Also, values for all particle size distribution parameters, including span, are in a wide range. This could be an indicator of a poorly conducted milling process which resulted in a final product with a very wide span of particle sizes in it, whose difference in size could cause flow difficulties and possible segregation during handling or storage [33]. Barbosa-Canovas et al. [28] showed in their work that the particle size of powder products produced by the milling process depends on the type of milling device used, the duration of milling and the properties of the materials being milled. In this study, the same milling device was used at the same duration; so, the properties of the materials remain the only factor that could lead to a wide range for all particle size distribution parameters. Also, according to the literature, moisture content also plays an important role in the milling process, whereby materials with a higher moisture content after the milling process is finished have a higher proportion of particles with larger diameters [34]. In this research, no clear connection was established between the moisture content in the sample and the size of the powder particles.

**Figure 1:**
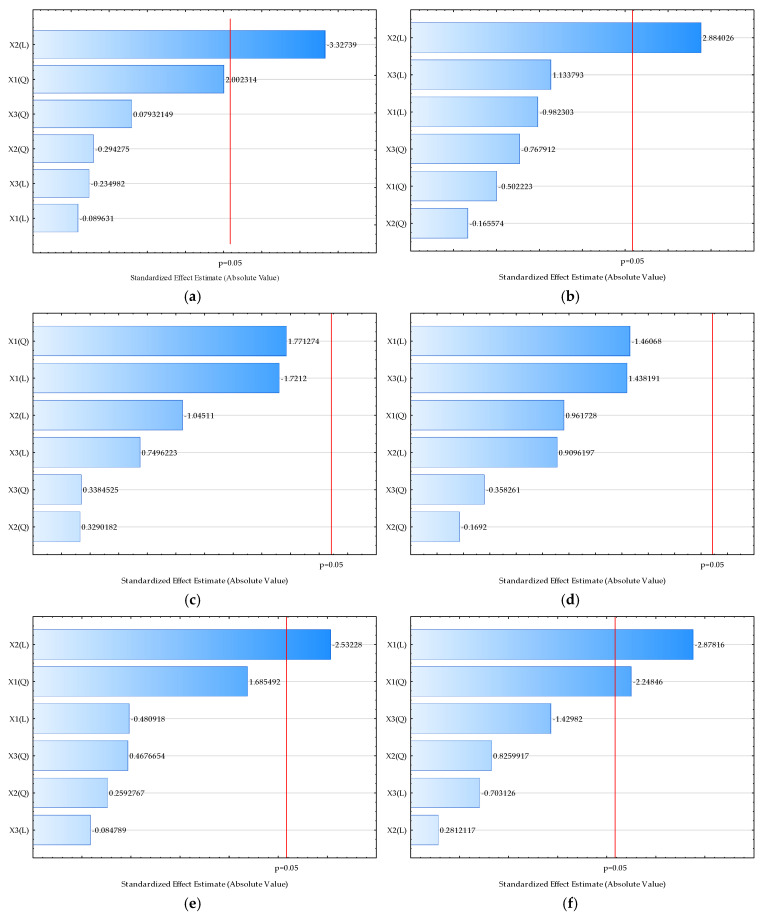

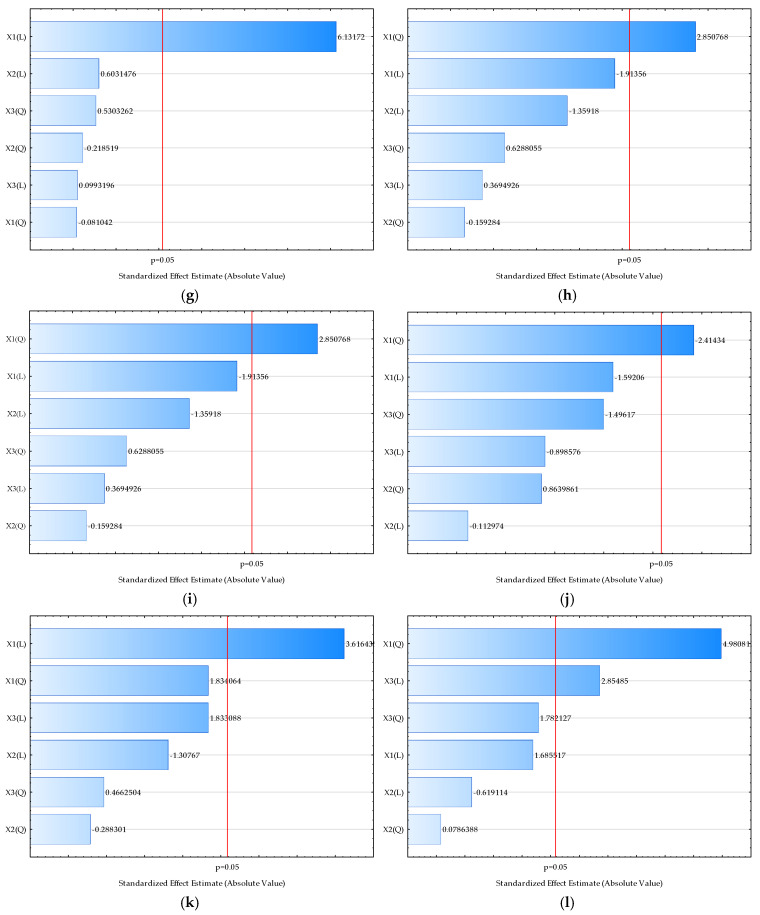

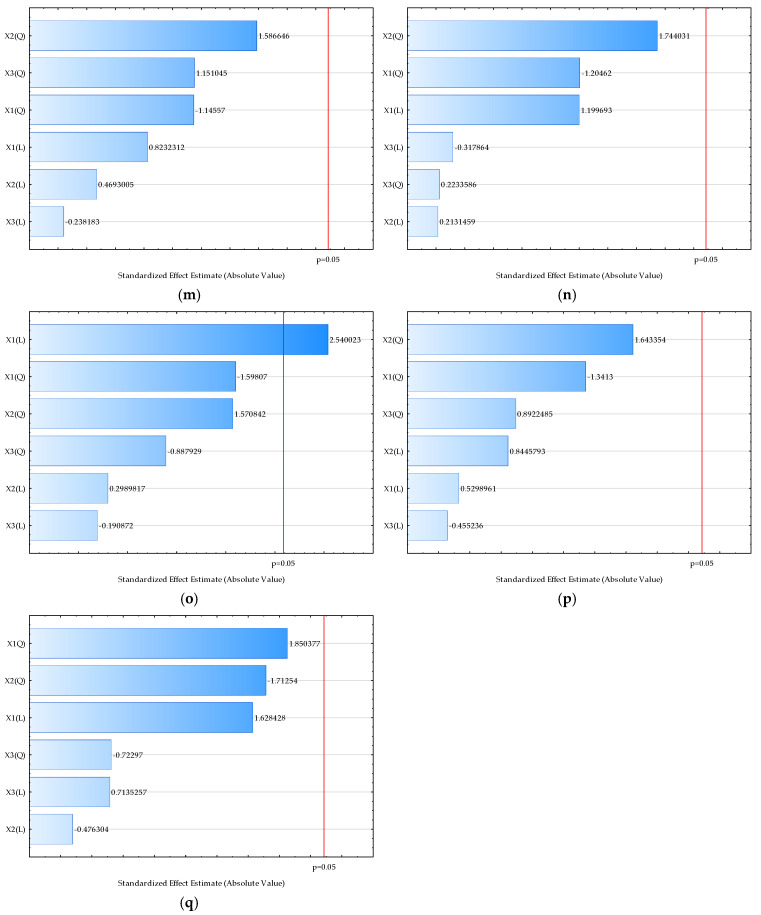
Pareto charts of standardized effect of the process variables on the selected outputs ((**a**) L*, (**b**) a*, (**c**) b*, (**d**) chroma, (**e**) hue, (**f**) moisture, (**g**) bulk density, (**h**) HR, (**i**) IC, (**j**) aw, (**k**) dispersibility, (**l**) wettability, (**m**) d (0.1), (**n**) d (0.5), (**o**) d (0.9), (**p**) D [3,2], (**q**) span). Values next to the bars represent the correlation coefficients. The letter “L” on the axis represents the linear coefficient of the model, while the letter “Q” represents the quadratic coefficient. The red line represents the probability level of *p* < 0.05.

To better understand the influence of input variables on physical properties, ANOVA was performed and the RSM models were developed based on the designed experiment. The results are shown as Pareto charts (Figure 1) and model equations describing the linear and quadratic effect of each input variable on the obtained results (Supplementary Table S1).

As seen in Figure 1a, a significant (*p* < 0.05) influence of the honey/oat flour ratio was detected on the L* values (brightness of the mixtures). The results showed a significant negative effect of the linear coefficient associated with the honey/oat flour ratio, indicating that a higher amount of oat in the mixture leads to increased brightness. A significant positive effect of the honey/oat flour ratio was also detected for a*, and a significant negative effect was observed for the hue color values (Figure 1c,e), while all other color parameters were not significantly influenced by the input process variables (Figure 1b,d).

A significant (*p* < 0.05) negative influence of temperature was detected on the moisture content (Figure 1f). The results showed a significant negative effect of both the linear and quadratic coefficients of the RSM model associated with temperature on the moisture content, indicating that a lower drying temperature results in a higher moisture content. A significant positive effect of temperature was also detected for values of bulk density, HR, CI, aw, and d (0.9) (Figure 1g–l,o). All effects were significant in the linear coefficient; for bulk density, HR, CI, and d (0.9), a higher temperature led to higher values of the enumerated properties, while for aw values, higher temperature resulted in lower values. There was no significant influence of input variables on d (0.1), d (0.5), D [3,2], and span (Figure 1m,n,p,q).

Buljat et al. [35] explored the possibility of foam mat drying for the production of instant cocoa powders enriched with lavender extracts by drying the foam at three different temperatures (T = 50, 60, and 70 °C). According to that study, samples dried at a lower temperature (T = 50 °C) exhibited the best powder flow and reconstitution properties.

According to the coefficients of determination for the RSM models developed (Supplementary Table S1), the best agreement between the experimental data and the model-predicted data was obtained for wettability (R^2^ = 0.6331), followed by bulk density (R^2^ = 0.6569). The results also showed that RSM models are not suitable for the prediction of cocoa powder mixture particle size. The coefficients of determination for the prediction of particle size distribution ranged from R^2^ = 0.2339 (model describing d (0.1)) to R^2^ = 0.3825 (model describing d (0.9)).

### 2.2. Chemical Properties of the Cocoa Mixture Powder Extracts

The chemical properties of the extracts (TDS (mg L^−1^), conductivity (µS cm^−1^), pH, Brix (°), L*, a*, b*, chroma and hue as color values, TPC (mg GAE gdm^−1^), DPPH (mmol TE gdm^−1^) and FRAP (mmol FeSO_4_ gdm^−1^) are presented in Table 2.

The results showed that for the cocoa mixture samples dried at T = 50 °C, the total dissolved solids (TDS) values ranged from 39.30 ± 0.92 mg L^−1^ (sample 50-7) to 64.30 ± 0.56 mg L^−1^ (sample 50-1), for samples dried at T = 60 °C, the TDS values ranged from 44.20 ± 0.95 mg L^−1^ (sample 60-5) to 69.90 ± 0.30 mg L^−1^ (sample 60-1), and for samples dried at T = 70 °C, the TDS values ranged from 51.03 ± 0.95 mg L^−1^ (sample 70-2) to 76.33± 0.01 mg L^−1^ (sample 70-1). Furthermore, as shown in Table 2, the conductivity values ranged from 75.87 ± 1.85 µS cm^−1^ (sample 50-7) to 128.67 ± 1.07 µS cm^−1^ (sample 50-1) for samples dried at T = 50 °C. For samples dried at T = 60 °C, the conductivity values ranged from 88.40 ± 1.91 µS cm^−1^ (sample 60-5) to 146.43 ± 0.97 µS cm^−1^ (sample 60-8), and for samples dried at T = 70 °C, the conductivity values ranged from 152.73 ± 0.30 µS cm^−1^ (sample 70-2) to 100.93 ± 1.01 µS cm^−1^ (sample 70-1). The TDS values were the highest in all samples containing 7.5 g of cocoa and with a ratio honey/oat flour 50:50 regardless of temperature, while the conductivity values were also the highest in all samples containing 7.5 g of cocoa and with a honey/oat flour ratio of 50:50, except for T = 60 °C, where the ratio honey/oat flour was of 40:60.

As shown in Table 2, for cocoa powders prepared by drying at T = 50 °C, the pH values of the extracts ranged from 6.09 ± 0.02 (sample 50-3, 50-7) to 6.36 ± 0.01 (sample 50-9), while for samples dried at T = 60 °C, the pH values ranged from 6.03 ± 0.02 (sample 60-6) to 6.27 ± 0.02 (sample 60-5), and for samples dried at T = 70 °C, the pH values ranged from 6.06 ± 0.02 (sample 70-4) to 6.78 ± 0.02 (sample 70-8). Higher values of pH were recorded in samples with a higher content of cocoa, while the ratio of honey/oat flour did not show an influence on the pH values of the extracts. As described by Puchol-Miquel et al. [36], according to the pH values, the cocoa product is labeled as dark natural if the pH is between 5.0 and 6.0, lightly alkalized cocoa when the pH is from 6.0 to 7.2, medium-alkalized cocoa when the pH range is between 7.2 and 7.6, and strongly alkalized cocoa when the product has a pH value higher than 7.6. Based on the listed categorizations, the cocoa powder produced in this work can be considered lightly alkalized.

For cocoa mixture samples dried at T = 50 °C, the total solid sugars expressed as Brix (°) of the extracts were ranged from 17.92 ± 0.14 °Bx (sample 50-7) to 19.50 ± 0.25 °Bx (sample 50-9). For extracts made of powder mixtures dried at T = 60 °C, Brix ranged from 20.33 ± 0.28 °Bx (sample 60-7) to 19.33 ± 0.14 °Bx (sample 60-5), and for extracts made of powder mixtures dried at T = 70 °C, the range was from 19.00 ± 0.43 °Bx (sample 70-3) to 20.33 ± 0.38 °Bx (sample 70-9). Samples with a higher honey content (ratio 60:40 and 50:50) showed higher °Bx values. However, cocoa powder generally does not contain sugar; therefore, the content of cocoa powder does not show a significant influence on the ° Bx values; in this study, higher °Bx values were measured on samples with a higher cocoa content.

As shown in Table 2, the color coordinate values of the extract were also measured. The L*-color coordinate values for extracts from samples dried at T = 50 °C ranged from 33.35 ± 0.38 (sample 50-1) to 37.19 ± 0.02 (sample 50-6), for extracts from samples dried at T = 60 °C, the L* value ranged from 40.45 ± 0.01 (sample 60-1) to 43.48 ± 0.01 (sample 60-9), and for extracts from samples dried at T = 70 °C, the L* value ranged from 44.04 ± 0.02 (sample 70-5) to 30.71 ± 0.09 (sample 70-4). The a*-color coordinate values for extracts from samples dried at T = 50 °C ranged from 0.67 ± 0.01 (sample 50-6) to 1.36 ± 0.01 (sample 50-8), for extracts from samples dried at T = 60 °C, the a* value ranged from 0.16 ± 0.01 (sample 60-5) to 0.95 ± 0.05 (sample 60-4), and for extracts from samples dried at T = 70 °C, the a* value ranged from −0.01 ± 0.02 (sample 70-2) to 1.44 ± 0.16 (sample 70-6). The b*-color coordinate values for extracts from samples dried at T = 50 °C ranged from 4.29 ± 0.01 (sample 50-7) to 5.70 ± 0.02 (sample 50-8), for extracts from samples dried at T = 60 °C, the b* value ranged from 4.25 ± 0.01 (sample 60-3) to 5.04 ± 0.01 (sample 60-4, 60-7), and for extracts from samples dried at T = 70 °C, the b* value ranged from 3.16 ± 0.17 (sample 70-3) to 5.15 ± 0.04 (sample 70-1). Chroma values for extracts from samples dried at T = 50 °C ranged from 4.38 ± 0.01 (sample 50-7) to 5.87 ± 0.02 (sample 50-8), for extracts from samples dried at T = 60 °C, the chroma value ranged from 4.27 ± 0.01 (sample 60-3) to 5.13 ± 0.01 (sample 60-4), and for extracts from samples dried at T = 70 °C, the chroma value ranged from 3.17 ± 0.17 (sample 70-3) to 5.39 ± 0.03 (sample 70-1). The hue value was also measured and for extracts from samples dried at T = 50 °C, it ranged from 53.64 ± 1.71 (sample 50-2) to 79.97 ± 0.20 (sample 50-9); for extracts from samples dried at T = 60 °C, the hue value ranged from 64.62 ± 0.08 (sample 60-3) to 87.86 ± 0.79 (sample 60-5), and for extracts from samples dried at T = 70 °C, the hue value ranged from 72.73 ± 0.17 (sample 70-1) to 90.22 ± 0.22 (sample 70-2).

The analysis of the color values L* (darkness to lightness), a* (redness to greenness), and b* (blueness to yellowness) after drying at different temperatures clearly shows the color change with respect to the L*, a*, and b* values. An increase in drying temperature results in an increase in the L* coordinate and a decrease in the a* and b* values. As previously described by Li et al. [37], high temperatures and high pH conditions favored the formation of dark components in cocoa powder. Maillard reactions under acidic or basic conditions produce a set of reaction products, including non-volatile colored compounds of intermediate molecular weight and brown substances of high molecular weight [38,39].

As previously described, the high polyphenol content of cocoa, combined with its wide presence in many food products, makes cocoa particularly interesting from a nutritional and health point of view [40]. For cocoa mixture samples dried at T = 50 °C, the total polyphenol content ranged from 2.617 ± 0.380 mg GAE g_dm_^−1^ (sample 50-6) to 4.061 ± 0.323 mg GAE g_dm_^−1^ (sample 50-2); for the cocoa mixture samples dried at T = 60 °C, it ranged from 3.670 ± 0.283 mg GAE g_dm_^−1^ (sample 60-2) to 2.474 ± 0.384 mg GAE g_dm_^−1^ (sample 60-1), and for the cocoa mixture samples dried at T = 70 °C, it ranged from 2.273 ± 0.107 mg GAE g_dm_^−1^ (sample 70-6) to 4.206 ± 0.135 mg GAE g_dm_^−1^ (sample 70-5). As cocoa powder is rich in polyphenols, the samples with a higher content of cocoa powder also had a higher content of total polyphenols. The values of total polyphenol content obtained are higher than those presented by Buljat et al. [35], where instant cocoa powdered enriched with lavender extract was prepared using foam mat drying. Furthermore, the results presented are more similar to those presented by Vieira de Oliveira [41], where the TPC of different commercial cocoa powders ranged from 1.117 to 4.126 GAE g_dm_^−1^.

Lee et al. [42], in their research, determined the content of polyphenols in cocoa powder and other foods that contain higher amounts of polyphenols. The results showed that cocoa powder contains 611 mg equivalent of gallic acid per serving (7.3 g of cocoa powder) and 564 mg equivalent of epicatechin per serving. It has also been determined that such gallic acid equivalent values are about 1.8; 3.7 and 4.9 times higher than the values obtained for red wine, green tea and black tea. The content of polyphenols can vary greatly depending on the source of the grain, the conditions of primary and secondary processing and the process of making chocolate. Because of these factors, it is unlikely that the ratio and types of polyphenols found in cocoa beans will be the same as those found in finished products. The alkalization of cocoa powder will reduce the polyphenol content and antioxidant activity [43].

From all of the powder samples presented in Table 1, ethanolic extracts (70% *w*/*w*) were made and their antioxidant capacity was determined by the DPPH method. For the extracts made from cocoa samples dried at T = 50 °C, the DPPH was in the range from 0.0115 ± 0.0015 mmol TE g_dm_^−1^ (sample 50-2) to 0.0238 ± 0.0005 mmol TE g_dm_^−1^ (sample 50-1); for the extracts made from cocoa samples dried at T = 60 °C, it was in the range from 0.0117 ± 0.0002 mmol TE g_dm_^−1^ (sample 60-9) to 0.2264 ± 0.0011 mmol TE g_dm_^−1^ (sample 60-4), and for the extracts made from cocoa samples dried at T = 70 °C, it was in the range from 0.0220 ± 0.0002 mmol TE g_dm_^−1^ (sample 70-6) to 0.0321 ± 0.0009 mmol TE g_dm_^−1^ (sample 70-5). The samples with a lower content of cocoa powder also had a lower antioxidant activity (as well as a lower content of polyphenols), while the samples with a higher content of cocoa powder also had a higher antioxidant activity (as well as a higher content of polyphenols) due to the fact that cocoa powder is rich in polyphenols and thus antioxidants. Jaćimović et al. [44] also stated that the samples with lower polyphenol and flavonoid content showed lower antioxidant activity. As for the influence of the honey/oat ratio on antioxidant activity, no clear trend was determined in this study.

Lee et al. [42], in their study, also determined that the value of the antioxidant activity of cocoa powder was measured by the DPPH method and it was 836 mg of ascorbic acid equivalent per serving (7.3 g); it was concluded that cocoa powder has a significant antioxidant capacity, 4-5 times stronger than black tea, 2–3 times stronger than green tea and 2 times stronger than red wine.

The antioxidant activity of the extracts was also determined by another method, the FRAP method, which is based on the reduction of the yellow-colored complex iron-2,4,6-tripyridyl-s-triazine (TPTZ), and the color of the solution changes to blue through the transition from the Fe^3+^ form of the complex to Fe^2+^ due to reduction by antioxidants For cocoa mixture samples dried at T = 50 °C, FRAP was in the range from 0.0248 ± 0.0007 mmol FeSO_4_ g_dm_^−1^ (sample 50-9) to 0.0333 ± 0.0001 mmol FeSO_4_ g_dm_^−1^ (sample 50-1); for the cocoa mixture samples dried at T = 60 °C, it was in the range from 0.0033 ± 0.0001 mmol FeSO_4_ g_dm_^−1^ (sample 60-2) to 0.0397 ± 0.0010 mmol FeSO_4_ g_dm_^−1^ (sample 60-9), and for the cocoa mixture samples dried at T = 70 °C, it was in the range from 0.0264 ± 0.0002 mmol FeSO_4_ g_dm_^−1^ (sample 70-6) to 0.0502 ± 0.0013 mmol FeSO_4_ g_dm_^−1^ (sample 70-5). As with the DPPH method, samples with a higher content of cocoa powder also showed a higher antioxidant activity by the FRAP method, while the ratio of honey/oats did not show a significant effect.

Carlsen et al. [45] determined the antioxidant activity of various products, including chocolate, using the FRAP method. What they concluded was that the mean proportion of antioxidants increases with the increase in cocoa content in the chocolate product, and their data show that chocolate products with a cocoa content of 24–30%, 40–65% and 70–99% had a mean antioxidant content of 1, 8, 7.2 and 10.9 mmol/100 g, respectively.

The reduction capacity of pure cocoa powder determined using the FRAP test was 9.38 mM Fe(II) for cocoa powder with 10–12% fat and 9.01 mM for cocoa powder with 16–18% fat in the research conducted by Belščak-Cvitanović et al. [29] because the FRAP values of the experimental mixtures ranged from 2.23 mM Fe(II) in the 10–12% fat mixture to 3.79 mM Fe(II) in the 16–18% fat mixture. According to the established results, the fat content in cocoa powder does not significantly (*p* > 0.05) affect the antioxidant capacity of the mixture of beverages with cocoa powder. According to experimental data, the phenolic content and antioxidant capacity of cocoa powder beverage mixtures should show approximately one third of the antioxidant capacity of pure cocoa liquor, as they contain 30% cocoa powder.

**Figure 2:**
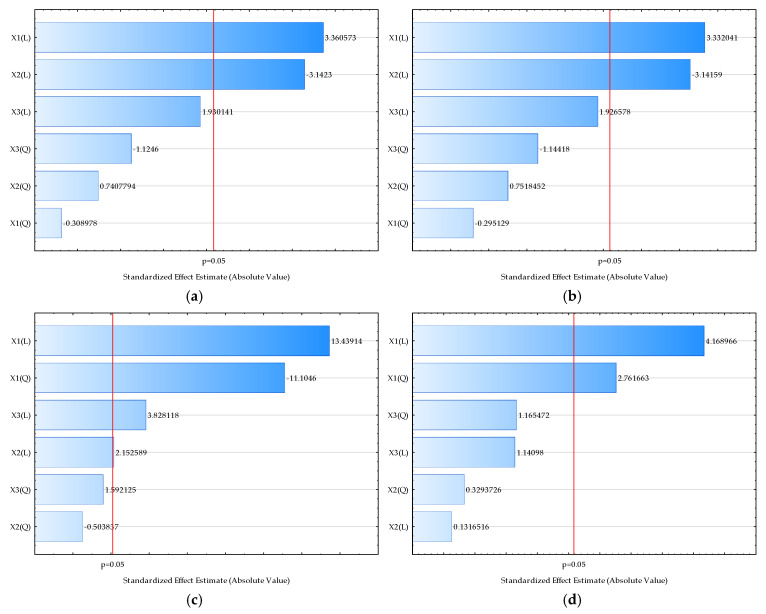

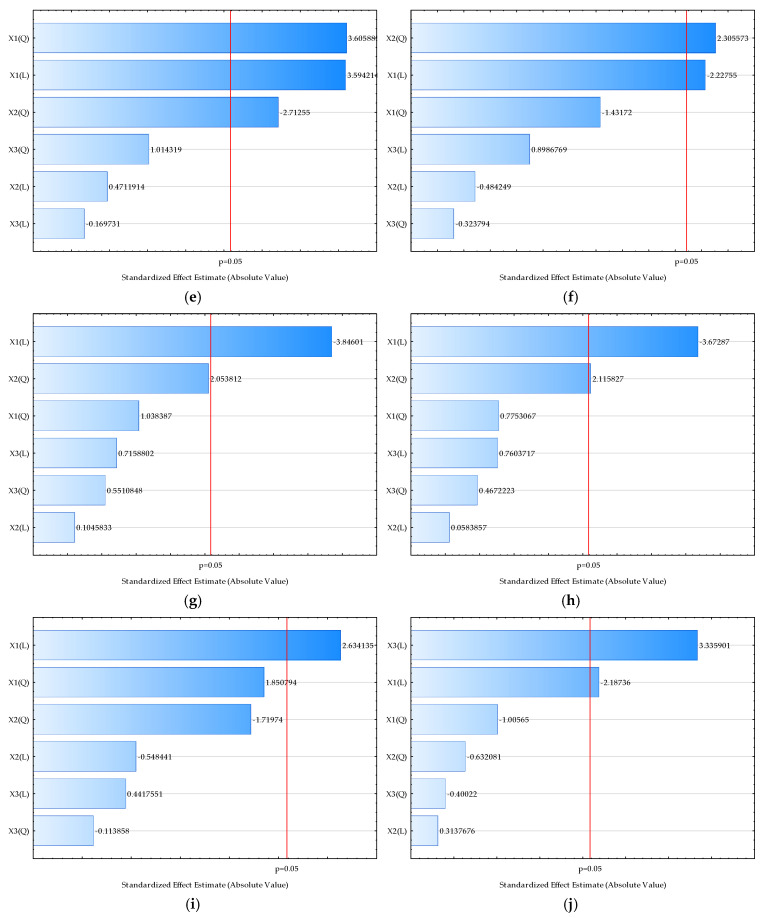

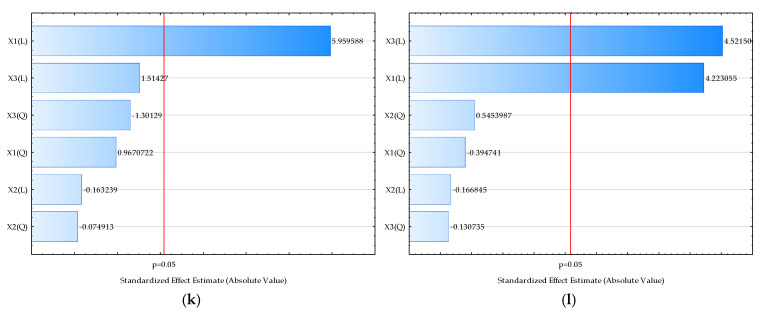
Pareto charts of standardized effect of the process variables on the selected outputs. (**a**) TDS, (**b**) conductivity, (**c**) pH, (**d**) Brix, (**e**) L*, (**f**) a*, (**g**) b*, (**h**) chroma, (**i**) hue, (**j**) TPC, (**k**) DPPH, (**l**) FRAP. Values next to the bars represent the correlation coefficients. The letter “L” on the axis represents the linear coefficient of the model, while the letter “Q” represents the quadratic coefficient. The red line represents the probability level of *p* < 0.05.

The estimation of significant influences of input variables on chemical properties is shown in the form of Pareto charts (Figure 2) and RSM models (Supplementary Table S2).

As seen from the Pareto charts, drying temperature has a significant positive effect on the TDS (Figure 2a), on the conductivity (Figure 2b), on the pH (Figure 2c), on the sugar content expressed as °Brix (Figure 2d), on the L*-coordinate of color (Figure 2e), the hue value of the color (Figure 2i), the TPC (Figure 2j), the DPPH (Figure 2k), and teh FRAP (Figure 2l). An increase in the drying temperature leads to lower values of b* and chroma (Figure 2g,h). The analysis of the standardized effect of the process variables on the selected outputs showed that both linear and quadratic coefficients of RSM models assessed with temperature have significant effects on the TDS (Figure 2a), conductivity (Figure 2b), pH (Figure 2c), sugar content expressed as °Brix (Figure 2d), and L-coordinate of color (Figure 2e). The standardized effect presented in the Pareto chart confirmed the previously described effect that drying temperature is the most important variable for the chemical properties of the cocoa powder extracts. The results also showed the significant negative effect of the honey/oat flour ratio on the TDS (Figure 2a), on the conductivity (Figure 2b), on the L*-coordinate of color (Figure 2e), and on the a* value of the color (Figure 2f). According to the results obtained, cocoa powder content has a significant positive effect on the pH (Figure 2c), on the TPC (Figure 2j), and on the FRAP (Figure 2l).

The simulations effects of the drying temperature, honey/oat flour ratio, and cocoa powder and on the analyzed chemical proprieties of the cocoa powder extract were analyzed using RSM modeling. The obtained model equations are given in Supplementary Table S2. The applicability of the developed models was estimated based on the coefficient of determination (R^2^) [46]. The best agreement between experimental data and model-predicted data [47] was obtained for the pH value (R^2^ = 0.9248), followed by DPPH (R^2^ = 0.6692) and FRAP (R^2^ = 0.6592). According to the results, the biggest dispersion between experimental data and model-predicted data was obtained for the RSM model describing the a*-coordinate of color (R^2^ = 0.4025).

### 2.3. Sensory Properties of Cocoa Mixture Powders

**Figure 3:**
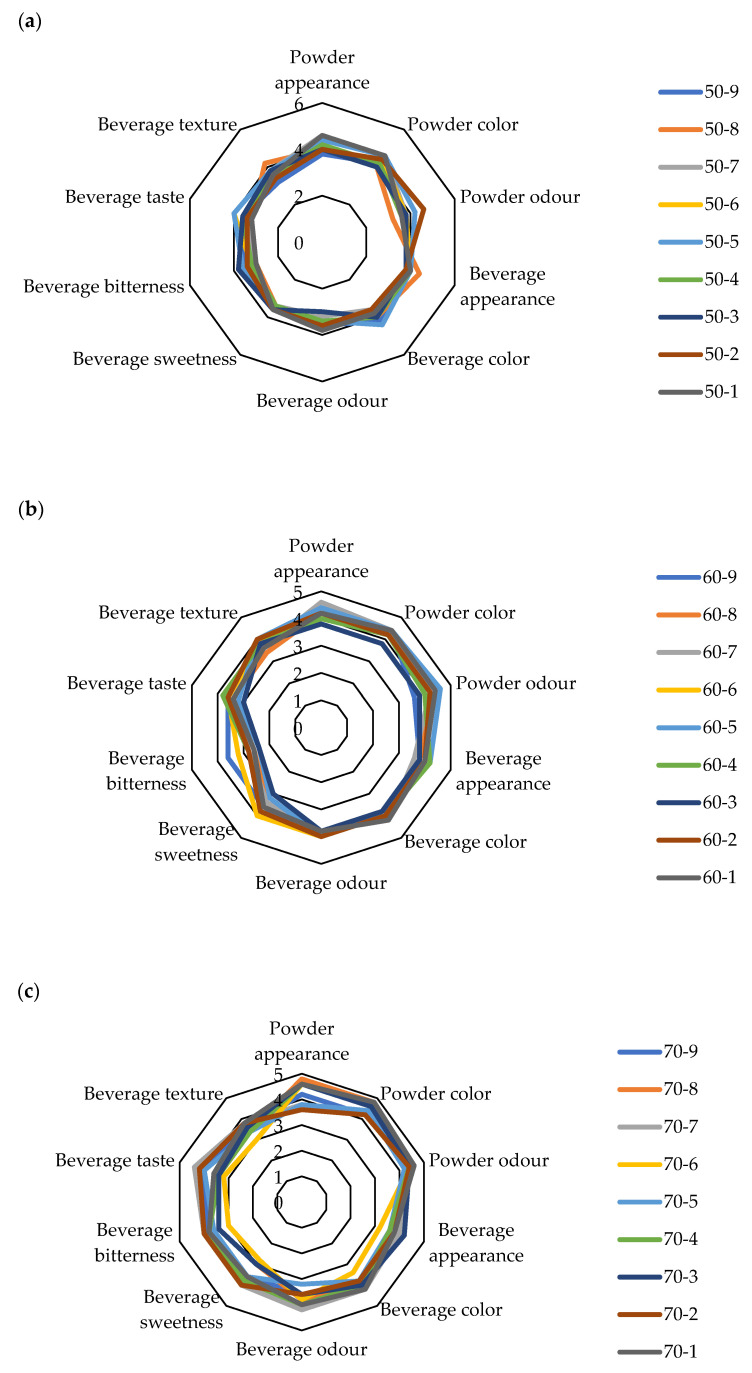
Sensory properties of analyzed samples (**a**) 50 °C, (**b**) 60 °C and (**c**) 70 °C.

The results for the sensory analysis of the powders (appearance, color, odor) and the prepared beverages (appearance, color, odor, sweetness, bitterness, taste, texture) are shown in Figure 3.

The sensory analysis of the cocoa powder and cocoa powder beverage involved evaluating the following aspects: the appearance, color, and odor of the powder, as well as the appearance, color, odor, sweetness, bitterness, taste, and texture of the beverage. Powder appearance included a visual estimation of the uniformity of particles and lump formation, and for the cocoa powders dried at T = 50 °C, the powder appearance property was graded from 3.8 (sample 50-9) to 4.6 (sample 50-1, 50-7), powder color was graded in a range from 4 (sample 50-3, 50-8) to 4.6 (sample 50-1, 50-5, 50-7), while the powder odor for samples dried at T = 50 °C were graded in a range from 3.2 (sample 50-8) to 4.6 (sample 50-2). The beverage appearance for beverages made of these mixtures was graded in a range from 3 (sample 50-3) to 3.8 (sample 50-1). The beverage color for the same sample was graded in a range from 3.6 (sample 50-2, 50-7) to 4.4 (sample 50-5), beverage odor was graded in a range from 3 (sample 50-3) to 3.8 (sample 50-1), and beverage sweetness was graded in a range from 3.4 (sample 50-4, 50-7, 50-8) to 3.6 for all other samples. Beverage bitterness for the same drying temperature was graded in a range from 3 (sample 50-1, 50-8) to 3.8 (sample 50-3), beverage taste was graded in a range from 3.2 (sample 50-1, 50-7, 50-8) to 4 (sample 50-5, 50-6), and beverage texture was graded from 3.6 (sample 50-1, 50-4) to 4.2 (sample 50-8).

For the cocoa powders dried at T = 60 °C, powder appearance was graded 3.8 (sample 60-3) to 4.6 (sample 60-7), and powder color was graded in a range from 3.8 (sample 60-3) to 4.4 (sample 60-1, 60-5, 60-6, 60-7, 60-9), while powder odor for samples dried at T = 60 °C was graded in a range from 3.6 (sample 60-9) to 4.6 (sample 60-5). The beverage appearance for beverages made of these mixtures was graded in a range from 3.8 (sample 60-3, 60-8, 60-9) do 4.2 (sample 60-4, 60-5). Beverage color for the same sample was graded in a range from 3.8 (sample 60-3, 60-5) to 4.2 (sample 60-1.60-7), beverage odor was graded in a range from 3.8 (sample 60-1, 60-3, 60-7) to 4 (all other samples), and beverage sweetness was graded in a range from 3 (sample 60-3) to 3.8 (sample 60-2, 60-4). Beverage bitterness for the same drying temperature was graded in a range from 2.4 (sample 60-3, 60-5) to 3.6 (sample 60-9), beverage taste was graded in a range from 3 (sample 60-3) to 3.8 (sample 60-4), and beverage texture was graded from 3.4 (sample 60-8) to 4 (sample 60-2, 60-5).

For the cocoa powders dried at T = 70 °C, powder appearance was graded in a range from 3.6 (sample 70-2) to 4.8 (sample 70-8), and powder color was graded in a range from 4.2 (sample 70-2, 70-7, 70-9) to 4.8 (sample 70-1, 70-8), while powder odor for samples dried at T = 70 °C was graded in a range from 4.2 (sample 70-5) to 4.6 (sample 70-1, 70-8). The beverage appearance for beverages made of these mixtures was graded in a range from 3.2 (sample 70-6) do 4.2 (sample 70-3). Beverage color for the same sample was graded in a range from 3.4 (sample 70-6) to 4.2 (sample 70-1, 70-7), beverage odor was graded in a range from 3.2 (sample 70-6) to 4.2 (sample 70-7), and beverage sweetness was graded in a range from 2.8 (sample 70-6) to 4 (sample 70-2, 70-7). Beverage bitterness for the same drying temperature was graded in a range from 3 (sample 70-6) to 4 (sample 70-2, 70-7), and beverage taste was graded in a range from 3.2 (sample 70-6) to 4.4 (sample 70-7). Beverage texture included a visual estimation of the presence of undissolved cocoa parts, as well as the in-mouth estimation of grittiness, viscosity and mouthfeel. Beverage texture was graded from 3 (sample 70-6) to 3.8 (sample 70-1, 70-2, 70-7).

According to the results, the appearance of the powders significantly depends on the powder color, while beverage taste increases with beverage sweetness. Similar results are presented in the work of Kowalska et al. [48], where the experts of sensory evaluation defined the cocoa powder samples containing 80% sucrose as the most perceptible and characteristic, mainly in terms of taste and aroma.

Belščak-Cvitanović et al. [29] also investigated the sensory properties of cocoa powder beverages with different characteristics. It was observed that the fat content of cocoa powder did not affect the sensory perception of consumers. With regard to the taste in the mouth and the sweetness and balance, mixtures with sugars (sucrose, glucose, trehalose) showed the advantage. The mix containing the sweetener aspartame/acesulfame K was rated the highest, but the mix containing stevia extract showed the most balanced properties with an average score of 6.14 determined by the panel. Such beverages were moderately sweet, had a good taste and provided a well-balanced taste, which indicates the great potential of using this sweetener.

**Figure 4:**
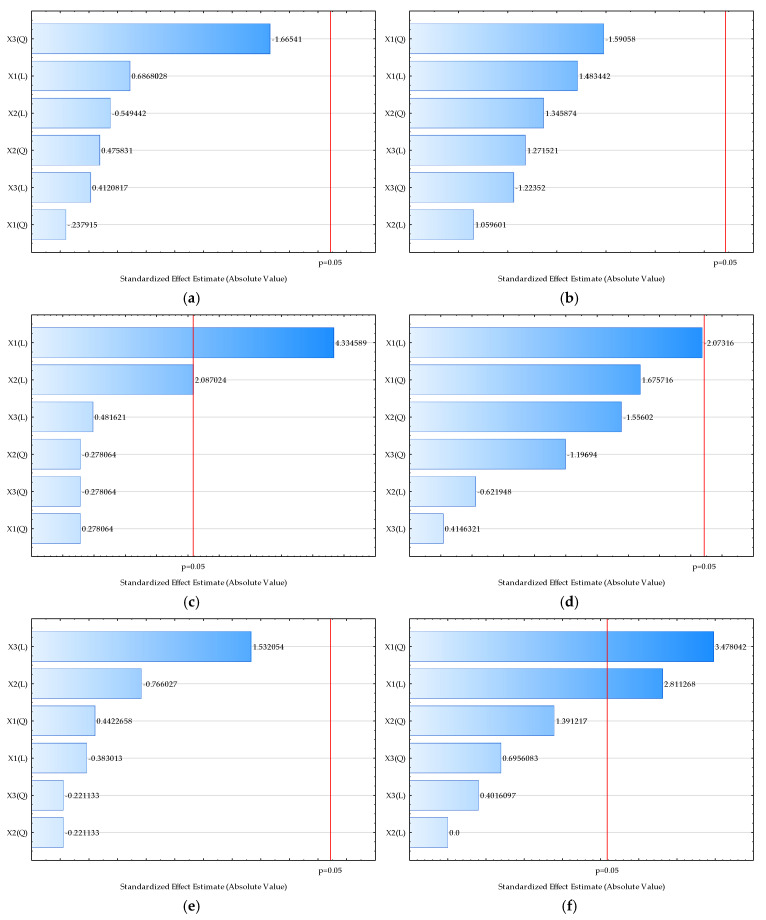

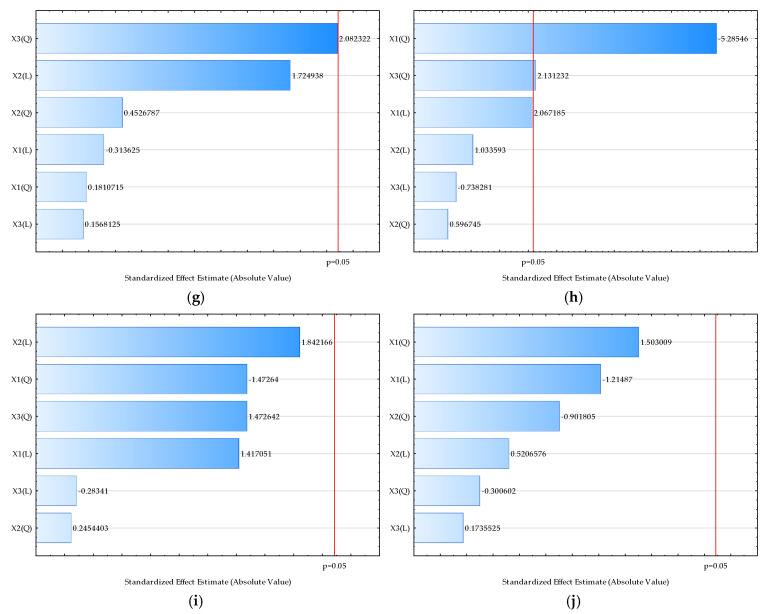
Pareto charts of standardized effect of the process variables on the selected outputs. (**a**) Powder appearance, (**b**) powder color, (**c**) powder odor, (**d**) beverage appearance, (**e**) beverage color, (**f**) beverage odor, (**g**) beverage sweetness, (**h**) beverage bitterness, (**i**) beverage taste, (**j**) beverage texture. Values next to the bars represent the correlation coefficients. The letter “L” on the axis represents the linear coefficient of the model, while the letter “Q” represents the quadratic coefficient. The red line represents the probability level of *p* < 0.05.

The influence of temperature, honey/oat flour and cocoa on sensory properties is shown in the form of Pareto charts (Figure 4) and correlation coefficients (Supplementary Table S3).

As shown in Figure 4c,f, a significant (*p* < 0.05) influence of temperature on powder odor and beverage odor was detected; a higher temperature leads to higher grades. Also, an increase in temperature leads to lower bitterness in beverages (Figure 4h). No other significant influence on outputs was detected (Figure 4a,b,d,e,g,i,j).

Al Aribah et al. [49], in their study, evaluated the impact of hydrocolloid incorporation on the quality attributes of the chocolate beverage. According to that study, there were no significant differences (*p* > 0.05) between chocolate beverages that added different types of hydrocolloids on the consistency, color, taste, and mouthfeel parameters, but by adding 0.2% of xanthan gum sensory, the values became higher in terms of consistency, color, aroma, taste, and mouthfeel.

RSM models were also applied for description and prediction of the sensory properties of both cocoa powders and cocoa powder beverages based on the drying temperature, honey/oat flour ratio, and amount of cocoa powder. According to coefficients of determination (Supplementary Table S3), the best agreement between experimental data and model-predicted data was obtained for the model describing the cocoa powder odor (R^2^ = 0.5414), while in the case of beverages, the highest value of the coefficient of determination (R^2^ = 0.6594) was obtained for the model describing beverage bitterness. Furthermore, according to the analysis of the standardized effects, temperature is the most important variable for the powder properties, while in the case of the beverage properties, the cocoa powder amount in the samples was also recognized as important.

### 2.4. Optimization of Process Conditions and Mixture Composition

The desirability profiling method was applied for process optimization, and the optimal values are presented in Table 3. The optimal process conditions were estimated specifically for the physical, chemical, and sensory properties of the analyzed samples using the desirability function. The desirability ranges from 0 (non-desirable) to 1 (highly desirable). Desirability was set to the maximum values for L*, a*, b*, chroma, and hue, while the lowest values of moisture, bulk density, HR, IC, aw, dispersibility, wettability, and all particle size distribution parameters were considered the most desirable. For chemical properties, desirability was set to the maximum. For sensory properties, desirability was also set to maximal values. According to the obtained results, the optimal process conditions for the physical properties of the samples are a drying temperature of 65 °C, a honey/oat flour ratio of 60%, and a cocoa content of 6.875 g/100 g. For the chemical properties, optimal process conditions are a drying temperature of T = 70 °C, a honey/oat flour ratio of 50%, and a cocoa content of 7.5 g/100 g, while for sensory properties, the optimal process conditions are a drying temperature of T = 70 °C, a honey/oat flour ratio of 60%, and a cocoa content of 7.5 g/100 g. The model-predicted values of the analyzed outputs for all three groups of the analyzed cocoa powder mixture properties are in the range of the experimentally obtained values. Furthermore, the results obtained show that each group of sample properties corresponds to specific process conditions.

## 3. Materials and Methods

### 3.1. Materials

The following materials were used in experiments: cocoa powder (10–12% fat) (Nutrigold, Zagreb, Croatia), refined oat flour (Nutrigold, Zagreb, Croatia), acacia honey (OPG Siniša Jurinjak, Krapina, Croatia), glyceryl monostearate (Elemental SRL, Oradea, Romania), arabic gum (Soul Food, Samobor, Croatia) and milk (2.8% milkfat) (Vindija, Varaždin, Croatia).

The chemicals used for the analyses were as follows: distilled water, ethanol (96%), (C_2_H_5_OH), (Kemika, Zagreb, Croatia); the Folin–Ciocalteu reagent (Supelco, Darmstadt, Germany), sodium carbonate (Na_2_CO_3_), (Gram-mol, Zagreb, Croatia); methanol (CH_3_OH), (Carlo Erba, Peypin, France); 1,1-diphenil-2-picrylhydrazyl (DPPH) (SigmaAldrich, Steinheim, Germany); hydrochloric acid (HCl), (Fischer Chemical, Loughborough, United Kingdom); sodium acetate-trihydrate (CH_3_COONa·3H_2_O), (J.T. Baker, Deventer, The Netherlands); acetic acid (CH_3_COOH), (T.T.T. d.o.o, Sveta Nedjelja, Croatia); 2,4,6-tripyridyl-1,3,5-triazine (TPTZ) (SigmaAldrich, Steinheim, Germany); iron (III)-chloride-hexahydrate (FeCl_3_·6H_2_O), (Gram-Mol, Zagreb, Croatia) and iron (II)-sulfate-heptahydrate (FeSO_4_·7H_2_O) (SigmaAldrich, Steinheim, Germany).

### 3.2. Methods

#### 3.2.1. Preparation and Drying of Mixtures

This research was conducted according to a full factorial experiment design with three parameters at three levels: (i) drying temperature (50, 60 and 70 °C); (ii) honey/oat flour ratio (40:60, 50:50 and 60:40); and (iii) the amount of cocoa powder (5, 6.25 and 7 g). The temperatures and the honey/oat ratios were chosen to ensure the stability of the bioactive components during drying and to avoid the glass transition of honey [15], while the cocoa amounts were adjusted based on preliminary sensory experiments to avoid the beverage being excessively bitter. According to the design of this experiment, 27 experiments were carried out according to different proportions of components in the mixtures and each mixture was dried at three temperatures (50 °C, 60 °C and 70 °C) (Table 4).

After weighing the cocoa powder, oat flour, honey, glyceryl monostearate (0.5 g per 100 g of the cocoa powder, oat flour, and honey mixture), and gum arabic (1 g per 100 g of the same mixture) based on the proportions defined by the experimental design (Table 1), water was added, and the ingredients were homogenized for 5 min using a stick mixer (Superior XB986F, Offenburg, Germany). The mixtures were then dried in a 31 × 21 cm metal container in a 10 mm thick layer in a convection oven (InkoLAB, Zagreb, Croatia) at 50 °C, 60 °C and 70 °C until a moisture content of 6 -10% was reached. After cooling, the mixtures were ground at 20,000 rpm for 1 min in an IKA Tube mill (IKA Werke, Staufen im Breisgau, Germany) to obtain a homogenous powder. The powders were stored at room temperature (20 °C) in sealed, opaque plastic containers and physical, chemical and sensorial analyses were carried out.

#### 3.2.2. Analysis of Physical Properties of Powders

##### Color Measurement

The color of the samples was measured by use of the PCE-CSM3 colorimeter (PCE Instruments, Southampton, UK), with prior calibration on a white plate. Five color parameters were determined: L* (light; L* =100 - white or L* = - black), a* (range from green (−a*) to red (+a*), b* (range from blue (−b*) to yellow (+b*)), chroma (color saturation) and hue value (the tone of the color) [50]. The color measurements were carried out in triplicate and the results are presented as the average value ± standard deviation.

##### Moisture Content

The moisture content of the powders was determined by drying the samples at 105 °C ± 2 °C for 3 h according to a standard AOAC method [51]. For each sample, measurements were carried out in triplicate and the results are presented as the average value ± standard deviation.

##### Bulk Density

Bulk density was determined according to a modified method previously described by Haugaard Sørensen et al. [52]. The cocoa powder mixtures were poured into a measuring cylinder, weighed and mounted on a laboratory made jolting volumeter. The measuring cylinder was subjected 0, 10,100 and 1250 taps and the bulk density was then calculated by dividing the mass of the powder by the volume recorded after 1250 taps. In order to minimize errors, the volume reading after 10 taps was chosen because of uneven distribution of the powder in the measuring cylinder immediately after pouring [53]. For each sample, measurements were performed in triplicate and the results are presented as the average value ± standard deviation.

From the measured values of free and tapped bulk density, two values can be calculated to characterize the flowability of powders: the Hausner ratio (*HR*) (Equation (1)) and the Carr index (*CI*) (Equation (2)) [28], where *ρ_free_* represents free bulk density (kg m^−^^3^) and *ρ_tapped_* represents tapped bulk density (kg m^−^^3^).

(1) $$HR=\frac{\rho_{tapped}}{\rho_{free}}$$

(2) $$CI=\frac{\rho_{tapped}-\rho_{free}}{\rho_{free}}\cdot 100$$

##### Water Activity

The water activity (aw) was determined using a Rotronic HygroPalm HP23 water activity device (Rotronic AG, Bassersdorf, Switzerland) by placing the sample in the measuring chamber and reading the value after the measurement was completed [54]. Measurements were performed in triplicate and the results are presented as the average value ± standard deviation.

##### Reconstitution Properties

Reconstitution properties were determined as dispersibility and wettability. Dispersibility was determined by a stirring test as the time in seconds taken to disperse a given amount of powder into a given amount of water of a given temperature [52]. One full teaspoon of a sample was dispersed in 100 mL of distilled water at room temperature. At the same time, a stopwatch was started and manual stirring began (25 circular stirring movements within 15 s) until all lumps were dispersed. Measurements were performed in triplicate and the results are presented as the average value ± standard deviation.

Wettability is defined as the time in seconds required for a given amount of powder to completely penetrate the still surface of a liquid. For the purposes of measurement, a paper funnel with a height of 100 mm, a lower diameter of 40 mm and an upper diameter of 90 mm was made into which the sample was poured before the measurement to ensure an even distribution of the powder on the liquid surface. The funnel with a pestle was placed on a beaker with 100 mL of distilled water at room temperature; one full teaspoon of powder was poured into the funnel, the pestle was removed and the stopwatch was started. Measurements were performed in triplicate and the results are presented as the average value ± standard deviation.

##### Particle Size Distribution

Particle size distribution was determined using the laser diffraction method with dry dispersion of the sample [55]. For each sample, measurements were performed using the Malvern Mastersizer 2000 with the Scirocco dry dispersion unit (Malvern Instruments, Malvern, UK) and the following parameters were determined: (i) d (0.1) particle size, which is smaller than 10% of particles of the entire sample; (ii) d (0.5) (mass median diameter) represents a diameter of particles compared to which 50% of the total number of particles have a larger or smaller diameter; (iii) d (0.9) particle size, which is smaller than 90% of the particles of the entire sample; (iv) D [3,2] surface weighted mean or Sauter mean diameter; (v) span as a measurement of the width of the distribution [33]. Measurements were carried out in triplicate and the results are presented as mean ± SD.

#### 3.2.3. Analysis of Chemical Properties of Extracts

##### Extract Preparation

The powder samples (3 g) were extracted using a 70% ethanol solution (90 mL) previously heated to 70 °C in a water/oil bath (IKA, Staufen, Germany). Ethanol was chosen as the extraction solvent due to its ability to sediment proteins [56]. The extraction was carried out for 30 min at a stirring speed of 500 rpm in covered glasses to prevent solvent evaporation. The obtained extract was filtered on a vacuum filtration set (Rocker 300-LF30, New Taipei City, Taiwan) and stored in 50 mL Falkon cuvettes. The samples were kept in the freezer for 24 h to enable protein sedimentation and centrifuged at 6000 rpm prior to the analyses. In that way, possible interference of protein components in chemical property analysis was prevented.

##### Total Dissolved Substance and Conductivity

Total dissolved solids (TDS) and conductivity were determined using a SevenCompact conductometer (Mettler Toledo, Columbus, OH, USA) by immersing the probe in the liquid extract [57]. The measurements were carried out in triplicate and results are presented as the average value ± standard deviation.

##### pH Value

The pH value of the extracts was determined by immersing a pH probe (Jenco 601A, Jenco Instrumens, San Diego, CA, USA) in the prepared extracts. The measurements were carried out in triplicate and results are presented as the average value ± standard deviation.

##### Sugar Content in Degrees Brix

Sugar content was determined using a refractometer (ABBE, 2WAJ, Bluewave Industry Co., Ltd., Shanghai, China) by the Brix method. Two drops of the sample extract were dropped on a measuring prism and the sugar content was read on the scale of the refractometer in the form of a refractive index and Brix (°) [58]. The measurements were carried out in triplicate and results are presented as the average value ± standard deviation.

##### Color Measurement

The color measurements were carried out as described in the section on physical properties. The measurements were carried out in triplicate and results are presented as the average value ± standard deviation.

##### Determination of Total Polyphenolic Content (TPC)

Total polyphenolic content (TPC) of the prepared extracts was determined spectrophotometrically by the Folin–Ciocalteu reagent, according to Singleton and Rossi [59] and Jurinjak Tušek et al. [60]. Distilled water (7.9 mL) was transferred to a glass test tube to which 100 μL of sample and 500 μL of the Folin-Ciocalteau reagent were added. The reaction began with the addition of 1.5 mL of 20% Na_2_CO_3_ solution and the samples were left to stand in the dark for 2 h. After 2 h, the absorbance at 765 nm was read using the Biochrom Libra 11 UV–vis spectrophotometer (Biochrom, Cambridge, UK). The measurements were carried out in triplicate and the results are expressed as mean values ± standard deviation of mg gallic acid equivalents (GAE) per gram of dry powder (mg GAE g_dm_^−^^1^), read from the gallic acid calibration curve.

##### Determination of Antioxidant Activity Using the DPPH Method

The antioxidant activity was determined spectrophotometrically based on the reaction of the tested sample and DPPH (2,2-diphenyl-1-picrylhydrazyl) methyl solution, which results in the discoloration of the solution. First, a 0.094 mM solution of DPPH radical in methanol was prepared. Then, 100 μL of sample was transferred to a glass test tube and 3.9 mL of 0.094 mM DPPH solution was added. The reaction mixture was then incubated at room temperature for 30 min in the dark. After 30 min, the absorbance at 515 nm was read using the Biochrom Libra 11 UV–vis spectrophotometer (Biochrom, Cambridge, UK) [61]. The measurements were carried out in triplicate and the results are expressed as mean values ± standard deviation of the molar fraction (mmol) of Trolox equivalents per gram of dry powder (mmol TE g_dm_^−^^1^), read from the Trolox calibration curve.

##### Determination of Antioxidant Activity Using the FRAP Method

Antioxidant activity was also determined by the FRAP (Ferric Reducing Antioxidant Power) method. The sample (50 μL) was transferred to a glass test tube to which 950 μL of the FRAP reagent was added. The reaction took place for 4 min in the dark, after which the absorbance at 593 nm was read using the Biochrom Libra 11 UV–vis spectrophotometer (Biochrom, Cambridge, UK) [62]. The measurements were carried out in triplicate and the results are expressed as mean values ± standard deviation of the molar equivalents of FeSO_4_·7H_2_O per gram of dry powder (mmol FeSO_4_ g_dm_^−^^1^).

#### 3.2.4. Sensory Analysis of Cocoa Powders and Drinks

Sensory analysis was performed by analysts (*n* = 10) according to the hedonistic scale. Scores 1-5 were assigned to samples depending on the property observed (score 1 meaning the sample in not acceptable, score 5 meaning high acceptability). Two groups of scores were given to the samples: the first scores were connected to the properties of the powders prior to beverage preparation (appearance (which includes uniformity of particles and lump formation), color, odor), while the second group of scores was given to hot beverages prepared by mixing 10 g of powder with 100 mL of milk and boiling the mixture for 3 min (appearance, color, odor, sweetness, bitterness, taste and texture (which included the presence of undissolved cocoa parts—grittiness, viscosity and mouthfeel)). The samples (V = 6 mL) were presented to analysts in a random order, in transparent plastic cups, together with the score sheet. Water was supplied to cleanse the pallet between tastings.

#### 3.2.5. Statistical Processing of Data and Optimization

Statistical analysis of the results obtained from the experiments was carried out using the software package Statistica v. 14 (Tibco Software, Palo Alto, USA). Basic statistical analysis included the calculation of the means and standard deviations, while the significant differences among the samples were tested using a *t*-test for independent samples at a probability level of *p* < 0.05. The design of this experiment was also made using the same software package, and to analyze the influence of independent variables (temperature, honey/oat ratio and cocoa contents) on all of the physical, chemical and sensory properties analyzed, ANOVA was performed, also at *p* < 0.05, and the results are shown as Pareto charts and model equations (Supplementary Files), according to Equation (3):

*Y* = *slope* + *A*· *X*_1_ + *B*· *X*_2_ + *C* · *X*_3_ + *D* · *X*_1_^2^ + *E* · *X*_2_^2^ + *F* · *X*_3_^2^ (3)

where *Y* represents the analyzed response, letters *A–E* represent the model generated regression coefficients (*A* – *C*—linear, *D* – *E*—quadratic), *X*_1_ represents the temperature (°C), *X*_2_ represents the honey/oat flour ratio (/), and *X*_3_ represents the proportion of cocoa powder (g).

Furthermore, mixture composition was optimized using the desirability profiling method, which was performed based on three different groups of properties: (i) drying temperature, honey/oat ratio and cocoa content were used as input variables, while the output variables were all the physical properties; (ii) drying temperature, honey/oat ratio and cocoa content were used as input variables, while the output variables were all the chemical properties; and (iii) drying temperature, honey/oat ratio and cocoa content were used as input variables, while the output variables were all the sensory properties [63].

## 4. Conclusions

In this study, the applicability of using honey as a sweetener and oat flour as a filler in a new cocoa powder mixture was confirmed. The good flowability, dispersibility, and wettability of the prepared cocoa powder mixtures makes them appealing to consumers. Furthermore, the TPC of the samples, which ranges from 2.273 ± 0.107 mg GAE g_dm_^−1^ to 4.206 ± 0.135 mg GAE g_dm_^−1^, contributes to the positive health effect of the prepared cocoa powder mixture. The results obtained indicate the impact of the systematic analysis of the process variables on the physical, chemical, and sensory properties of the newly developed cocoa powder mixtures. To elucidate the intricate interactions between components within the formulated mixtures, future investigations should focus on identifying specific chemical modifications that occur during the manufacturing process. Additionally, exploring the potential influence of oat components as fillers on the bioavailability of bioactive compounds in cocoa and honey would provide valuable insights into the overall nutritional and functional properties of the final product.

## Figures and Tables

**Table 1:** Physical properties of powder mixtures shown as mean value ± SD (*n* = 3) *.

Sample	L*	a*	b*	Chroma	Hue	Moisture (%)	Bulk Density (kgm^−3^)	HR	CI	Water Activity	Dispersibility (s)	Wettability (s)	d (0.1) (µm)	d (0.5) (µm)	d (0.9) (µm)	D [3,2] (µm)	Span
50-1	37.94 ± 0.02 ^a^	16.48 ± 0.01 ^a^	19.20 ± 0.01 ^a^	25.31 ± 0.02 ^a^	49.36 ± 0.01 ^a^	11.32 ± 0.03 ^a^	666.49 ± 33.32 ^a^	1.14 ± 0.06 ^a^	14.45 ± 0.72 ^a^	0.51 ± 0.004 ^a^	8.88 ± 1.06 ^a^	16.63 ± 0.73 ^a^	94.22 ± 10.20 ^a^	304.86 ± 37.10 ^a^	663.03 ± 73.33 ^a^	186.30 ± 19.9 ^a^	1.87 ± 0.03 ^a^
50-2	31.23 ± 0.01 ^b^	15.54 ± 0.01 ^b^	14.69 ± 0.01 ^b^	21.38 ± 0.02 ^b^	43.3 ± 0.00 ^b^	11.13 ± 0.08 ^b^	720.55 ± 36.03 ^b^	1.18 ± 0.06 ^a^	18.52 ± 0.93 ^b^	0.49 ± 0.003 ^b^	7.73 ± 0.54 ^b^	24.75 ± 1.92 ^b^	169.48 ± 59.62 ^b^	470.11 ± 95.97 ^b^	889.76 ± 112.38 ^b^	313.48 ± 90.1 ^b^	1.53 ± 0.19 ^b^
50-3	56.55 ± 0.25 ^c^	13.64 ± 0.03 ^c^	18.76 ± 0.04 ^c^	23.2 ± 0.05 ^c^	53.97 ± 0.01 ^c^	6.95 ± 0.06 ^c^	627.96 ± 31.39 ^c^	1.24. ± 0.06 ^b^	24.09 ± 1.20 ^c^	0.35 ± 0.005 ^c^	11.32 ± 1.37 c	81.74 ± 7.66 ^c^	26.43 ± 1.10 ^c^	219.56 ± 6.35 ^c^	572.38 ± 22.10 ^c^	64.80 ± 1.73 ^c^	2.49 ± 0.48 ^c^
50-4	50.42 ± 0.01 ^d^	14.98 ± 0.06 ^d^	20.11 ± 0.02 ^d^	25.12 ± 0.01 ^d^	53.31 ± 0.02 ^d^	8.34 ± 0.07 ^d^	672.54 ± 33.63 ^a^	1.18. ± 0.06 ^a^	17.95 ± 1.20 ^b^	0.38 ± 0.005 ^d^	11.12 ± 0.84 ^c^	27.21 ± 2.81 ^d^	92.97 ± 0.80 a	303.26 ± 6.17 ^a^	665.44 ± 20.92 ^a^	184.88 ± 1.66 ^a^	1.89 ± 0.48 ^a^
50-5	34.89 ± 0.01 ^e^	16.1 ± 0.01 ^e^	16.9 ± 0.01 ^e^	23.41 ± 0.01 ^e^	46.34 ± 0.02 ^e^	7.75 ± 0.12 ^e^	641.27 ± 32.06 ^a^	1.21 ± 0.06 ^b^	20.18 ± 1.01 ^d^	0.36 ± 0.001 ^e^	8.25 ± 0.11 ^a^	27.86 ± 4.87 ^d^	174.07 ± 5.66 ^b^	407.06 ± 13.28 ^b^	795.96 ± 21.58 ^d^	319.31 ± 9.10 ^b^	1.53 ± 0.02 ^b^
50-6	42.99 ± 0.01 ^f^	15.03 ± 0.01 ^f^	18.92 ± 0.01 ^f^	24.16 ± 0.01 ^f^	51.54 ± 0.01 ^f^	11.33 ± 0.10 ^f^	640.36 ± 32.02 ^a^	1.88 ± 0.09 ^c^	18.82 ± 0.94 ^b^	0.49 ± 0.002 ^f^	7.66 ± 0.51 ^b^	14.27 ± 2.57 ^a^	126.11 ± 2.57 ^d^	336.39 ± 5.91 ^d^	741.47 ± 10.43 ^d^	238.49 ± 4.04 ^d^	1.83 ± 0.01 ^a^
50-7	43.99 ± 0.02 ^g^	14.58 ± 0.01 ^g^	18.59 ± 0.01 ^g^	23.63 ± 0.01 ^g^	51.9 ± 0.03 ^g^	9.07 ± 0.08 ^g^	692.79 ± 34.64 ^a^	1.21 ± 0.06 ^b^	20.99 ± 1.05 ^d^	0.41 ± 0.002 ^g^	5.44 ± 0.51 ^d^	11.22 ± 1.38 ^e^	98.85 ± 3.09 ^g^	376.94 ± 9.77 ^e^	858.17 ± 16.55 ^b^	206.65 ± 1.57 ^e^	2.01 ± 0.02 ^d^
50-8	49.95 ± 0.01 ^h^	15.32 ± 0.006 ^h^	20.06 ± 0.01 ^h^	25.24 ± 0.05 ^h^	52.64 ± 0.01 ^h^	9.60 ± 0.08 ^h^	701.98 ± 35.10 ^a^	1.12 ± 0.06 ^a^	18.60 ± 0.93 ^b^	0.44 ± 0.01 ^h^	16.44 ± 1.19 ^e^	63.17 ± 2.74 ^f^	66.50 ± 3.31 ^e^	450.58 ± 8.88 ^f^	906.71 ± 45.41 ^b^	126.63 ± 68.17 ^f^	1.86 ± 0.20 ^a^
50-9	39.89 ± 0.05 ^i^	14.85 ± 0.04 ^i^	18.25 ± 0.01 ^i^	23.53 ± 0.01 ^i^	50.85 ± 0.09 ^i^	10.21 ± 0.01 ^i^	650.00 ± 32.50 ^a^	1.21 ± 0.06 ^b^	20.99 ± 1.05 ^d^	0.47 ± 0.005 ^i^	8.40 ± 0.65 ^a^	21.43 ± 1.28 ^g^	139.99 ± 2.50 ^d^	339.98 ± 9.79 ^e^	707.34 ± 36.39 d	259.66 ± 3.26 ^g^	1.67 ± 0.08 ^b^
60-1	48.65 ± 0.02 ^j^	15.04 ± 0.01 ^j^	19.31 ± 0.01 ^j^	24.47 ± 0.01 ^j^	52.08 ± 0.01 ^j^	6.51 ± 0.09 ^j^	724.09 ± 36.20 ^b^	1.19 ± 0.06 ^a^	18.75 ± 0.37 ^b^	0.32 ± 0.004 ^j^	27.19 ± 9.08 ^f^	261.08 ± 12.75 ^h^	37.90 ± 0.26 ^f^	238.06 ± 2.09 ^g^	612.82 ± 9.11 ^a^	81.86 ± 0.41 ^h^	2.41 ± 0.04 ^c^
60-2	50.93 ± 0.01 ^d^	14.42 ± 0.02 ^k^	19.36 ± 0.01 ^k^	24.14 ± 0.01 ^k^	53.32 ± 0.03 ^d^	5.35 ± 0.06 ^k^	700.63 ± 35.03 ^a,b^	1.23 ± 0.06 ^b^	23.39 ± 1.17 ^c^	0.26 ± 0.001 ^k^	24.87 ± 2.45 ^g^	239.87 ± 14.99 ^i^	29.35 ± 0.44 ^g^	202.23 ± 4.40 ^h^	569.40 ± 23.06 ^c^	69.09 ± 0.88 ^i^	2.67 ± 0.05 c
60-3	44.25 ± 0.03 ^k^	14.22 ± 0.01 ^l^	17.71 ± 0.01 ^l^	22.71 ± 0.01 ^l^	51.24 ± 0.03 ^k^	9.64 ± 0.03 ^h^	706.67 ± 35.03 ^b^	1.17 ± 0.06 ^a^	17.56 ± 0.35 ^b^	0.47 ± 0.007 ^l^	11.32 ± 1.13 ^c^	53.30 ± 9.67 ^j^	71.84 ± 2.21 ^e^	327.41 ± 15.81 ^e^	840.94 ± 76.76 ^b^	147.28 ± 4.77 ^j^	2.33 ± 0.11 ^c^
60-4	50.83 ± 0.01 ^d^	14.34 ± 0.16 ^m^	19.41 ± 0.01 ^m^	24.19 ± 0.01 ^m^	53.36 ± 0.03 ^l^	6.11 ± 0.03 ^l^	689.84 ± 34.49 ^a,b^	1.24 ± 0.06 ^b^	24.41 ± 1.22 ^c^	0.31 ± 0.004 ^m^	24.44 ± 1.95 ^g^	150.16 ± 15.77 ^k^	31.33 ± 1.57 ^g^	204.47 ± 10.23 ^h^	514.53 ± 27.73 ^e^	69.56 ± 3.48 ^k^	2.36 ± 0.56 ^c^
60-5	46.65 ± 0.01 ^l^	14.9 ± 0.01 ^n^	19.24± 0.01 ^n^	24.33 ± 0.01 ^n^	52.24 ± 0.02 ^m^	6.84 ± 0.11 ^m^	695.53 ± 34.78 ^a,b^	1.24 ± 0.06 ^b^	24.03 ± 0.48 ^c^	0.33 ± 0.001 ^n^	15.59 ± 1.62 ^e^	184.28 ± 16.69 ^l^	41.40 ± 1.60 ^h^	230.64 ± 11.47 ^g^	678.49 ± 34.92 ^a^	92.93 ± 3.90 ^l^	2.76 ± 0.25 ^c^
60-6	42.67 ± 0.03 ^m^	14.55 ± 0.02 ^o^	17.85 ± 0.03 ^o^	23.14 ± 0.22 ^c^	50.82 ± 0.04 ^i^	9.24 ± 0.04 ^n^	719.45 ± 35.97 ^b^	1.21 ± 0.06 ^b^	20.63 ± 1.03 ^d^	0.45 ± 0.004 ^o^	11.60 ± 1.56 ^c^	43.22 ± 5.69 ^m^	183.51 ± 9.17 ^b^	792.73 ± 39.64 ^i^	1454.75 ± 72.74 ^f^	362.58 ± 18.13 ^m^	1.60 ± 0.09 ^b^
60-7	35.68 ± 0.01 ^n^	17.83 ± 0.02 ^p^	18.31 ± 0.03 ^p^	25.56 ± 0.03 ^o^	45.76 ± 0.02 ^n^	9.33 ± 0.07 ^o^	721.65 ± 36.08 ^b^	1.17 ± 0.06 ^a^	16.92 ± 0.85 ^b^	0.45 ± 0.002 ^p^	10.70 ± 0.88 ^c^	30.17 ± 5.64 ^d^	144.04 ± 13.83 ^b^	409.00 ± 38.12 ^b^	927.72 ± 47.39 ^b^	278.81 ± 25.40 ^g^	1.92 ± 0.07 ^a^
60-8	51.09 ± 0.04 ^d^	13.87 ± 0.01 ^r^	18.63 ± 0.01 ^r^	23.23 ± 0.01 ^c^	53.34 ± 0.02 ^o^	5.43 ± 0.05 ^p^	684.73 ± 34.24 ^a,b^	1.26 ± 0.06 ^b^	25.60 ± 1.28 ^c^	0.26 ± 0.001 ^q^	32.11 ± 4.96 ^h^	247.71 ± 19.24 ^i^	16.01 ± 0.82 ^i^	185.62 ± 7.73 ^j^	514.80 ± 35.49 ^e^	47.48 ± 1.54 ^n^	2.69 ± 0.09 ^c^
60-9	54.74 ± 0.13 ^o^	14.17 ± 0.02 ^s^	19.01 ± 0.025 ^s^	23.71 ± 0.03 ^p^	53.28 ± 0.02 ^p^	4.45 ± 0.01 ^q^	675.04 ± 33.75 ^a,d^	1.26 ± 0.06 ^b^	26.56 ± 1.33 ^c^	0.23 ± 0.003 ^r^	22.95 ± 3.52 ^g^	201.91 ± 21.76 ^n^	19.27 ± 0.33 ^j^	201.76 ± 20.93 ^h^	560.63 ± 52.89 ^c^	52.89 ± 0.49 ^o^	2.68 ± 0.02 ^c^
70-1	44.68 ± 0.01 ^p^	14.56 ± 0.01 ^n^	17.07 ± 0.01 ^t^	22.44 ± 0.02 ^q^	49.55 ± 0.01 ^q^	7.14 ± 0.05 ^r^	736.37 ± 36.82 ^e^	1.23 ± 0.06 ^b^	22.69 ± 1.13 ^c^	0.37 ± 0.002 ^s^	16.12 ± 6.80 ^e^	84.78 ± 12.32 ^c^	91.53 ± 4.58 ^a^	535.43 ± 26.77 ^b^	1379.74 ± 431.67 ^f^	172.62 ± 14.63 ^p^	2.41 ± 0.10 ^c^
70-2	41.51 ± 0.01 ^r^	15.58 ± 0.01 ^g^	18.39 ± 0.01 ^u^	24.10 ± 0.01 ^r^	49.67 ± 0.08 ^r^	7.01 ± 0.04 ^s^	725.14 ± 36.26 ^a,c^	1.12 ± 0.06 ^b^	12.31 ± 0.61 ^e^	0.36 ± 0.001 ^t^	14.97 ± 5.16 ^e^	115.40 ± 24.29 ^o^	65.01 ± 5.57 ^e^	290.79 ± 35.36 ^a^	818.80 ± 149.08 ^b^	141.18 ± 12.00 ^j^	2.59 ± 0.17 ^c^
70-3	47.39 ± 0.02 ^s^	14.48 ± 0.13 ^k^	17.21 ± 0.16 ^v^	22.49 ± 0.21 ^s^	49.92 ± 0.01 ^s^	7.00 ± 0.07 ^c^	716.63 ± 35.83 ^a,c^	1.21 ± 0.06 ^b^	21.01 ± 1.05 ^c^	0.38 ± 0.001 ^u^	33.64 ± 5.29 ^h^	69.78 ± 8.99 ^f^	71.53 ± 10.07 ^e^	369.89 ± 81.69 ^e^	958.29 ± 142.13 ^b^	132.16 ± 6.62 ^k^	2.40 ± 0.11 ^c^
70-4	48.42 ± 0.01 ^t^	13.97 ± 0.01 ^t^	17.29 ± 0.01 ^w^	22.24 ± 0.01 ^t^	51.06 ± 0.02 ^t^	6.25 ± 0.05 ^t^	769.75 ± 38.49 ^a,b,c^	1.21 ± 0.06 ^b^	20 ± 1.00 ^c^	0.35 ± 0.001 ^c^	17.54 ± 3.08 ^e^	11.77 ± 16.06 ^e^	49.38 ± 3.07 ^k^	274.81 ± 17.38 ^k^	750.28 ± 95.31 ^d^	95.86 ± 4.72 ^l^	2.55 ± 0.17 ^c^
70-5	38.3 ± 0.02 ^u^	16.15 ± 0.01 ^u^	18.01± 0.01 ^x^	26.18 ± 1.72 ^a,d,h^	49.67 ± 0.00 ^u^	7.69 ± 0.22 ^e^	742.74 ± 37.14 ^a,b^	1.35 ± 0.07 ^d^	13.49 ± 0.67 ^a^	0.39 ± 0.001 ^v^	33.71 ± 5.07 ^h^	55.73 ± 35.88 ^j^	76.19 ± 19.90 ^e^	346.80 ± 91.05 ^e^	1021.62 ± 282.02 ^b^	161.71 ± 9.13 ^p^	2.73 ± 0.08 ^c^
70-6	42.38 ± 0.01 ^v^	14.41 ± 0.01 ^k^	16.89 ± 0.03 ^y^	22.21 ± 0.01 ^u^	49.56 ± 0.02 ^v^	7.42 ± 0.01 ^u^	755.54 ± 37.77 ^a,c^	1.14 ± 0.06 ^a^	14.4 ± 0.72 ^a^	0.39 ± 0.001 ^w^	15.91 ± 5.35 ^e^	34.85 ± 6.48 ^d^	83.33 ± 1.63 ^l^	363.59 ± 10.70 ^e^	935.677 ± 38.06 ^b^	169.65 ± 2.93 ^p^	2.35 ± 0.11 ^c^
70-7	41.34 ± 0.03 ^x^	14.41 ± 0.01 ^k,^	17.26 ± 0.01 ^z^	22.48 ± 0.05 ^v^	50.13 ± 0.02 ^w^	6.82 ± 0.01 ^m^	729.29 ± 36.46 ^a,c^	1.33 ± 0.07 ^d^	13.28 ± 0.66 ^a^	0.35 ± 0.002 ^c^	13.14 ± 3.01 ^e^	23.52 ± 2.09 ^b^	88.18 ± 5.06 ^l^	339.06 ± 20.80 ^e^	801.867 ± 35.06 ^b^	181.35 ± 10.16 ^a^	2.10 ± 0.07 ^e^
70-8	39.81 ± 0.01 ^y^	14.64 ± 0.01 ^v^	16.71 ± 0.01 ^q^	22.22 ± 0.01 ^w^	48.78 ± 0.02 ^x^	9.75 ± 0.22 ^h^	727.53 ± 36.37 ^a,c^	1.15 ± 0.06 ^a^	14.84 ± 0.74 ^a^	0.49 ± 0.001 ^e^	14.69 ± 1.57 ^e^	42.81 ± 3.58 ^m^	126.38 ± 6.32 ^d^	536.66 ± 26.83 ^l^	1305.77 ± 97.36 ^f^	238.68 ± 13.92 ^d^	2.20 ± 0.25 ^e^
70-9	41.95 ± 0.01 ^z^	14.93 ± 0.01 ^m^	18.25 ± 0.57 ^g^	23.23 ± 0.17 ^c^	50.21 ± 0.06 ^y^	7.23 ± 0.03 ^v^	751.01 ± 37.55 ^a,b,c^	1.14 ± 0.06 ^a^	14.52 ± 0.73 ^a^	0.38 ± 0.005 ^x^	27.38 ± 4.55 ^f^	116.87 ± 20.42 ^o^	922.78 ± 61.51 ^m^	1279.54 ± 24.30 ^m^	1653.99 ± 34.19 ^g^	1091.32 ± 98.58 ^q^	0.57 ± 0.07 ^f^

* Different letters above the number in the same column represent significant differences at *p* < 0.05.

**Table 2:** Chemical properties cocoa mixture powder extracts shown as mean value ± SD (*n* = 3) *.

Sample	TDS (mg L^−1^)	Conductivity (µS cm^−1^)	pH	Brix (°)	L*	a*	b*	Chroma	Hue	TPC (mg GAE g_dm_^−1^)	DPPH (mmol TE g_dm_^−1)^	FRAP (mmol FeSO_4_ g_dm_^−1^)
50-1	64.30 ± 0.56 ^a^	128.67 ± 1.07 ^a^	6.30 ± 0.01 ^a^	18.42 ± 0.14 ^a^	33.35 ± 0.38 ^a^	1.28 ± 0.06 ^a^	4.59 ± 0.04 ^a^	4.76 ± 0.50 ^a^	74.44 ± 0.68 ^a^	3.383 ± 0.563 ^a^	0.0238 ± 0.0005 ^a^	0.0333 ± 0.0001 ^a^
50-2	52.63 ± 0.99 ^b^	105.23 ± 2.03 ^b^	6.28 ± 0.01 ^b^	19.08 ± 0.52 ^b^	36.45 ± 0.03 ^b^	1.22 ± 0.05 ^a^	5.28 ± 0.01 ^b^	5.42 ± 0.01 ^b^	53.64 ± 1.71 ^b^	4.061 ± 0.323 ^a^	0.0115 ± 0.0015 ^b^	0.0281 ± 0.0004 ^b^
50-3	57.97 ± 2.06 ^c^	115.73 ± 3.93 ^c^	6.09 ± 0.02 ^c^	18.92 ± 0.38 ^c^	34.43 ± 0.02 ^c^	0.96 ± 0.06 ^b^	4.77 ± 0.01 ^c^	4.87 ± 0.01 ^a^	78.59 ± 0.08 ^c^	3.452 ± 0.215 ^a^	0.0153 ± 0.0007 ^c^	0.0282 ± 0.0001 ^b^
50-4	63.23 ± 1.35 ^a^	126.43 ± 2.65 ^a^	6.23 ± 0.01 ^d^	18.75 ± 0.25 ^c^	35.22 ± 0.01 ^d^	1.08 ± 0.06 ^c^	4.78 ± 0.01 ^c^	4.91 ± 0.01 ^a^	77.17 ± 0.06 ^c^	3.052 ± 0.313 ^b^	0.0180 ± 0.0017 ^d^	0.0242 ± 0.0001 ^c^
50-5	56.30 ± 1.04 ^c^	112.60 ± 2.08 ^c^	6.34 ± 0.06 ^e^	19.33 ± 0.14 ^b^	34.88 ± 0.08 ^e^	1.12 ± 0.02 ^c^	5.16 ± 0.06 ^d^	5.25 ± 0.12 ^c^	77.79 ± 0.11 ^c^	3.759 ± 0.528 ^a^	0.0192 ± 0.0004 ^d^	0.0300 ± 0.0001 ^d^
50-6	56.87 ± 0.32 ^c^	113.70 ± 2.07 ^c^	6.11 ± 0.01 ^c^	19.00 ± 0.25 ^b^	37.19 ± 0.02 ^f^	0.67 ± 0.01 ^d^	4.55 ± 0.01 ^a^	4.60 ± 0.01 ^d^	57.29 ± 0.14 ^d^	2.617 ± 0.380 ^c^	0.0142 ± 0.0009 ^e^	0.0262 ± 0.0002 ^e^
50-7	39.30 ± 0.92 ^d^	75.87 ± 1.85 ^d^	6.09 ± 0.02 ^c^	17.92 ± 0.14 ^d^	35.61 ± 0.01 ^g^	0.86 ± 0.02 ^d^	4.29 ± 0.01 ^e^	4.38 ± 0.01 ^e^	78.53 ± 0.32 ^c^	2.736 ± 0.302 ^c^	0.0147 ± 0.0022 ^e^	0.0228 ± 0.0001 ^f^
50-8	55.57 ± 1.45 ^c^	111.10 ± 2.95 ^c^	6.18 ± 0.01 ^f^	18.75 ± 0.43 ^c^	36.03 ± 0.13 ^h^	1.36 ± 0.01 ^f^	5.70 ± 0.02 ^f^	5.87 ± 0.02 ^f^	76.52 ± 0.07 ^d^	3.914 ± 0.289 ^a^	0.0169 ± 0.0004 ^f^	0.0322 ± 0.0004 ^a^
50-9	45.03 ± 1.19 ^e^	90.00 ± 2.33 ^e^	6.36 ± 0.01 ^e^	19.50 ± 0.25 ^b^	35.18 ± 0.10 ^d^	0.87 ± 0.03 ^d^	4.49 ± 0.12 ^a^	5.01 ± 0.13 ^g^	79.97 ± 0.20 ^c^	3.626 ± 0.237 ^a^	0.0146 ± 0.0007 ^e^	0.0248 ± 0.0007 ^g^
60-1	69.90 ± 0.30 ^f^	139.83 ± 0.60 ^f^	6.10 ± 0.02 ^c^	20.08 ± 0.72 ^e^	40.45 ± 0.01 ^i^	0.79 ± 0.01 ^e^	4.65 ± 0.01 ^g^	4.72 ± 0.01 ^a^	80.25 ± 0.09 ^e^	3.670 ± 0.283 ^a^	0.0183 ± 0.0002 ^g^	0.0391 ± 0.0002 ^h^
60-2	51.43 ± 0.31 ^g^	102.9 ± 0.70 ^b^	6.19 ± 0.02 ^f^	19.67 ± 0.14 ^b^	40.91 ± 0.06 ^j^	0.57 ± 0.01 ^f^	4.48 ± 0.04 ^a^	4.51 ± 0.04 ^h^	82.79 ± 0.07 ^f^	2.474 ± 0.384 ^c^	0.0224 ± 0.0001 ^h^	0.0332 ± 0.0001 ^a^
60-3	56.43 ± 0.21 ^c^	112.83 ± 0.40 ^c^	6.16 ± 0.01 ^g^	19.92 ± 0.29 ^e^	42.45 ± 0.01 ^k^	0.40 ± 0.01 ^g^	4.25 ± 0.01 ^h^	4.27 ± 0.01 ^i^	64.62 ± 0.08 ^g^	2.792 ± 0.256 ^c^	0.0273 ± 0.0001 ^i^	0.0241 ± 0.0001 ^c^
60-4	66.77 ± 0.68 ^h^	133.53 ± 1.36 ^g^	6.06 ± 0.02 ^h^	19.83 ± 0.14 ^e^	41.66 ± 0.01 ^l^	0.95 ± 0.05 ^b^	5.04 ± 0.01 ^i^	5.13 ± 0.01 ^j^	79.34 ± 0.05 ^e^	3.055 ± 0.054 ^b^	0.0226 ± 0.0011 ^h^	0.0310 ± 0.0002 ^i^
60-5	44.20 ± 0.95 ^e^	88.40 ± 1.91 ^h^	6.27 ± 0.02 ^b^	20.33 ± 0.28 ^e^	42.58 ± 0.02 ^k^	0.16 ± 0.01 ^h^	4.29 ± 0.12 ^e^	4.29 ± 0.12 ^i^	87.86 ± 0.79 ^h^	3.151 ± 0.326 ^b^	0.0234 ± 0.0007 ^a^	0.0295 ± 0.0001 ^j^
60-6	60.17 ± 0.23 ^i^	120.30 ± 0.43 ^i^	6.03 ± 0.02 ^i^	20.08 ± 0.14 ^e^	42.48 ± 0.01 ^k^	0.73 ± 0.01 ^i^	4.76 ± 0.01 ^c^	4.81 ± 0.01 ^a^	81.25 ± 0.11 ^e^	2.685 ± 0.162 ^c^	0.0267 ± 0.0015 ^j^	0.0276 ± 0.0001 ^k^
60-7	53.27 ± 0.15 ^b^	106.53 ± 0.38 ^b^	6.13 ± 0.01 ^c^	19.33 ± 0.14 ^b^	42.24 ± 0.01 ^k^	0.83 ± 0.01 ^j^	5.04 ± 0.01 ^i^	5.10 ± 0.01 ^j^	80.59 ± 0.13 ^e^	2.795 ± 0.188 ^c^	0.0249 ± 0.0005 ^k^	0.0285 ± 0.0001 ^b^
60-8	73.20 ± 0.43 ^j^	146.43 ± 0.97 ^j^	6.09 ± 0.01 ^c^	19.58 ± 0.14 ^e^	41.39 ± 0.01 ^l^	0.67 ± 0.01 ^d^	4.53 ± 0.01 ^a^	4.58 ± 0.02 ^d^	81.63 ± 0.05 ^e^	3.198 ± 0.290 ^b^	0.0219 ± 0.0004 ^l^	0.0311 ± 0.0001 ^i^
60-9	58.33 ± 3.76 ^c^	116.73 ± 7.54 ^c^	6.07 ± 0.01 ^h^	19.33 ± 0.14 ^b^	43.48 ± 0.01 ^k^	0.84 ± 0.01 ^j^	4.72 ± 0.01 ^c^	4.79 ± 0.01 ^a^	79.92 ± 0.09 ^c^	3.284 ± 0.176 ^b^	0.0117 ± 0.0002 ^b^	0.0385 ± 0.0010 ^j^
70-1	76.33 ± 0.01 ^k^	152.73 ± 0.30 ^k^	6.63 ± 0.03 ^j^	19.25 ± 0.29 ^b^	33.09 ± 0.05 ^a^	1.60 ± 0.01 ^k^	5.15 ± 0.04 ^d^	5.39 ± 0.03 ^b^	72.73 ± 0.17 ^i^	3.278 ± 0.175 ^b^	0.0312 ± 0.0022 ^m^	0.0398 ± 0.0018 ^j^
70-2	51.03 ± 0.95 ^g^	100.93 ± 1.01 ^l^	6.73 ± 0.06 ^k^	20.17 ± 0.14 ^e^	45.24 ± 0.03 ^m^	-0.01 ± 0.02 ^l^	3.67 ± 0.01 ^j^	3.67 ± 0.01 ^k^	90.22 ± 0.22 ^j^	2.438 ± 0.094 ^c^	0.0242 ± 0.0001 ^k^	0.0312 ± 0.0001 ^i^
70-3	66.80 ± 0.15 ^h^	133.70 ± 0.36 ^g^	6.51 ± 0.01 ^l^	19.00 ± 0.43 ^c^	42.07 ± 0.05 ^k^	0.03 ± 0.35 ^m^	3.16 ± 0.17 ^k^	3.17 ± 0.17 ^l^	84.53 ± 0.65 ^k^	2.903 ± 0.054 ^c^	0.0224 ± 0.0002 ^l^	0.0339 ± 0.0010 ^a^
70-4	74.93 ± 0.06 ^k^	149.23 ± 0.12 ^m^	6.06 ± 0.02 ^h^	19.75 ± 0.43 ^e^	43.72 ± 0.02 ^k^	0.04 ± 0.01 ^m^	3.43 ± 0.01 ^l^	3.47 ± 0.01 ^m^	83.27 ± 1.81 ^k^	3.041 ± 0.093 ^b^	0.0270 ± 0.0009 ^j^	0.0375 ± 0.0023 ^l^
70-5	73.10 ± 0.47 ^j^	146.23 ± 7.05 ^m^	6.70 ± 0.01 ^k^	19.50 ± 0.50 ^b^	44.04 ± 0.02 ^n^	0.65 ± 0.01 ^d^	3.51 ± 0.03 ^m^	3.57 ± 0.03 ^n^	79.54 ± 0.15 ^c^	4.206 ± 0.135 ^d^	0.0321 ± 0.0009 ^m^	0.0502 ± 0.0013 ^m^
70-6	64.87 ± 0.21 ^a^	129.77 ± 0.50 ^a^	6.56 ± 0.02 ^m^	19.50 ± 0.50 ^b^	30.71 ± 0.09 ^o^	1.44 ± 0.16 ^f^	4.95 ± 0.36 ^n^	5.16 ± 0.36 ^j^	73.71 ± 2.07 ^i^	2.273 ± 0.107 ^c^	0.0220 ± 0.0002 ^l^	0.0264 ± 0.0002 ^e^
70-7	64.10 ± 0.17 ^a^	128.13 ± 0.32 ^a^	6.60 ± 0.01 ^j^	19.75 ± 0.43 ^e^	42.87 ± 0.05 ^k^	0.43 ± 0.01 ^n^	3.45 ± 0.01 ^l^	3.47 ± 0.01 ^m^	82.85 ± 0.11 ^k^	3.106 ± 0.187 ^b^	0.0235 ± 0.0009 ^a^	0.0282 ± 0.0007 ^b^
70-8	64.97 ± 0.31 ^a^	129.90 ± 0.53 ^a^	6.78 ± 0.02 ^n^	20.33 ± 0.29 ^e^	42.17 ± 0.02 ^k^	0.34 ± 0.01 ^o^	3.26 ± 0.03 ^k^	3.28 ± 0.03 ^n^	83.97 ± 0.14 ^k^	2.757 ± 0.249 ^c^	0.0268 ± 0.0010 ^j^	0.0433 ± 0.0026 ^n^
70-9	56.13 ± 1.99 ^c^	112.27 ± 3.97 ^c^	6.71 ± 0.03 ^k^	20.33 ± 0.38 ^e^	37.75 ± 0.28 ^f^	0.91 ± 0.01 ^b^	4.77 ± 0.02 ^c^	4.86 ± 0.02 ^a^	79.18 ± 0.11 ^c^	2.673 ± 0.525 ^c^	0.0277 ± 0.0018 ^i^	0.0364 ± 0.0005 ^l^

* Different letters above the number in the same column represent significant differences at *p* < 0.05.

**Table 3:** Optimal process parameters and model-predicted values of physical, chemical and sensory properties at optimal conditions ^$^.

Optimal Process Parameters for Physical Properties		
T (°C)	Oat/honey ratio (%)	Cocoa content (g/100 g)
65	60	6.875
Model-predicted values of physical properties at optimal conditions		
Property	Value	Confidence interval (95%)
L*	42.80	37.93–47.66
a*	15.34	14.48–16.11
b*	18.15	17.08–19.21
Chroma	24.03	22.93–25.13
Hue	49.86	47.69–52.03
Moisture (%)	6.22	4.75–7.69
Bulk density (kg m^−3^)	727.68	704.92–750.44
HR	1.19	1.16–1.23
IC	19.64	16.37–22.90
a_w_	0.31	0.25–0.37
Dispersibility (s)	21.86	15.66–28.06
Wettability (s)	165.75	118.24–213.27
d (0.1) (µm)	110.24	45.08–265.56
d (0.5) (µm)	327.44	122.42–532.47
d (0.9) (µm)	779.68	537.05–1022.31
D [3,2] (µm)	183.66	13.96–353.37
span	2.42	1.99–2.85
Optimal process parameters for chemical properties		
T (°C)	Oat/honey ratio (%)	Cocoa content (g/100 g)
70	50	7.5
Model-predicted values of chemical properties at optimal conditions		
Property	Value	Confidence interval (95%)
TDS (mg L^−1^)	71.55	64.02–79.08
Conductivity (µS cm^−1^)	143.09	127.98–158.21
pH	6.68	6.61–6.76
Brix (°)	19.82	19.34–20.30
L*	37.58	34.56–40.60
a*	0.99	0.62–1.36
b*	4.28	3.71–4.84
Chroma	4.41	3.79–5.02
Hue	78.74	71.51–85.97
TPC (mg GAE g_dm_^−1^)	3.25	2.80–3.70
DPPH (mmol TE g_dm_^−1^)	0.03	0.02–0.03
FRAP (mmol FeSO_4_ g_dm_^−1^)	0.04	0.04–0.05
Optimal process parameters for sensory properties		
T (°C)	Oat/honey ratio (%)	Cocoa content (g/100 g)
70	60	7.5
Model-predicted values of sensory properties at optimal conditions		
Property	Value	Confidence interval (95%)
Powder–appearance	4.33	3.97–4.70
Powder–color	4.61	4.37–4.84
Powder–odour	4.62	4.31–4.93
Beverage–appearance	3.81	3.56–4.05
Beverage–color	3.99	3.73–4.25
Beverage–odour	3.71	3.46–3.96
Beverage–sweetness	3.52	3.20–3.84
Beverage–bitterness	3.55	3.21–3.89
Beverage–taste	3.80	3.45–4.15
Beverage–texture	3.62	3.33–3.91

^$^ Desirability was set to maximum values for L*, a*, b*, chroma and hue, while the lowest values of moisture, bulk density, HR, IC, aw, dispersibility, wettability and all particle size distribution parameters were considered the most desirable. For chemical properties, desirability was set to maximum. For sensory properties, desirability was also set to maximal values.

**Table 4:** Full factorial experiment design for the preparation of cocoa powder mixtures (3 factors at 3 levels—temperature (X_1_) (50, 60 and 70 °C), honey/oat ratio (X_2_) (60:40, 50:50 and 40:60) and cocoa contents (X_3_) (5, 6.25 and 7.5 g)). Values in brackets represent coded values.

Sample	X_1_ Temperature (°C)	X_2_ Honey/Oat Flour Ratio (%)	X_3_ Cocoa Powder Contents (g)
50-1	50 (−1)	50:50 (0)	7.50 (+1)
50-2	50 (−1)	60:40 (+1)	6.25 (0)
50-3	50 (−1)	40:60 (−1)	5.00 (−1)
50-4	50 (−1)	40:60 (−1)	6.25 (0)
50-5	50 (−1)	60:40 (+1)	7.50 (+1)
50-6	50 (−1)	50:50 (0)	5.00 (−1)
50-7	50 (−1)	60:40 (+1)	5.00 (−1)
50-8	50 (−1)	40:60 (−1)	7.50 (+1)
50-9	50 (−1)	50:50 (0)	6.25 (0)
60-1	60 (0)	50:50 (0)	7.50 (+1)
60-2	60 (0)	60:40 (+1)	6.25 (0)
60-3	60 (0)	40:60 (−1)	5.00 (−1)
60-4	60 (0)	40:60 (−1)	6.25 (0)
60-5	60 (0)	60:40 (+1)	7.50 (+1)
60-6	60 (0)	50:50 (0)	5.00 (−1)
60-7	60 (0)	60:40 (+1)	5.00 (−1)
60-8	60 (0)	40:60 (−1)	7.50 (+1)
60-9	60 (0)	50:50 (0)	6.25 (0)
70-1	70 (+1)	50:50 (0)	7.50 (+1)
70-2	70 (+1)	60:40 (+1)	6.25 (0)
70-3	70 (+1)	40:60 (−1)	5.00 (−1)
70-4	70 (+1)	40:60 (−1)	6.25 (0)
70-5	70 (+1)	60:40 (+1)	7.50 (+1)
70-6	70 (+1)	50:50 (0)	5.00 (−1)
70-7	70 (+1)	60:40 (+1)	5.00 (−1)
70-8	70 (+1)	40:60 (−1)	7.50 (+1)
70-9	70 (+1)	50:50 (0)	6.25 (0)

## Data Availability

Data available on request from the authors.

## Supplementary Materials

The following supporting information can be downloaded at: https://www.mdpi.com/article/10.3390/molecules29194665/s1: Supplementary Tables S1–S3. Table S1. RSM models for description of physical properties of powder mixtures (X_1_ temperature, X_2_ honey oat flour ratio, X_3_ proportion of cocoa powder); Table S2. RSM models for description of chemical properties of extracts (X_1_ temperature, X_2_ honey oat flour ratio, X_3_ proportion of cocoa powder); Table S3. RSM models for description of sensory properties of powders and beverages (X_1_ temperature, X_2_ honey oat flour ratio, X_3_ proportion of cocoa powder).
