# Supplementary material for: Development of Novel Honey- and Oat-Based Cocoa Beverages—A Comprehensive Analysis of the Impact of Drying Temperature and Mixture Composition on Physical, Chemical and Sensory Properties
Source: Molecules. 2024 Sep 30;29(19):4665. doi: 10.3390/molecules29194665 (PMC11477636; doi:10.3390/molecules29194665)
Supplement: Supplementary file 1 [file molecules-29-04665-s001.zip › Supplementary Table S1.pdf]

Supplementary Table S1. RSM models for description of physical properties of powder mixtures (X_1 temperature, X_2 honey oat flour ratio, X_3 proportion of cocoa powder).

	Model equation	R^2
**L***	$Y = - 110.82 + 5.148 \cdot X_1 - 104.433 \cdot X_2 + 13.392 \cdot X_3 - 0.043 \cdot X_1^2 + 63.185 \cdot X_2^2 - 1.09 \cdot X_3^2$	0.4422
**a***	$Y = 25.7901 - 0.2219 \cdot X_1 + 0.0315 \cdot X_2 - 1.8918 \cdot X_3 + 0.0017 \cdot X_1^2 + 5.5741 \cdot X_2^2 + 0.1654 \cdot X_3^2$	0.3638
**b***	$Y = - 15.7226 + 0.9545 \cdot X_1 + 12.6574 \cdot X_2 + 1.4387 \cdot X_3 - 0.0083 \cdot X_1^2 - 15.5 \cdot X_2^2 - 0.102 \cdot X_3^2$	0.2851
**Chroma**	$Y = 12.5465 - 0.0046 \cdot X_1 - 5.6574 \cdot X_2 - 1.0675 \cdot X_3 - 0.0046 \cdot X_1^2 + 8.2037 \cdot X_2^2 + 0.1112 \cdot X_3^2$	0.2341
**Hue**	$Y = - 14.462 + 1.9092 \cdot X_1 + 10.8222 \cdot X_2 + 3.5233 \cdot X_3 - 0.0161 \cdot X_1^2 - 24.8148 \cdot X_2^2 - 0.2865 \cdot X_3^2$	0.3283
**Moisture (%)**	$Y = 76.272 - 1.8577 \cdot X_1 + 54.6255 \cdot X_2 - 7.6295 \cdot X_3 + 0.0146 \cdot X_1^2 - 53.5725 \cdot X_2^2 + 0.5935 \cdot X_3^2$	0.4541
**Bulk density ($kg\ m^{-3}$)**	$Y = 422.34 + 2.579 \cdot X_1 - 184.509 \cdot X_2 + 43.075 \cdot X_3 + 0.008 \cdot X_1^2 + 219.486 \cdot X_2^2 - 3.409 \cdot X_3^2$	0.6569
**HR**	$Y = - 0.2875 + 0.0477 \cdot X_1 - 0.3427 \cdot X_2 + 0.0745 \cdot X_3 - 0.0004 \cdot X_1^2 + 0.2296 \cdot X_2^2 - 0.0058 \cdot X_3^2$	0.4151
**IC**	$Y = - 128.748 + 4.772 \cdot X_1 - 34.373 \cdot X_2 + 7.497 \cdot X_3 - 0.041 \cdot X_1^2 + 22.961 \cdot X_2^2 - 0.58 \cdot X_3^2$	0.2396
**Water activity**	$Y = 3.408 - 0.0827 \cdot X_1 + 2.3736 \cdot X_2 - 0.3428 \cdot X_3 + 0.0007 \cdot X_1^2 - 2.3916 \cdot X_2^2 + 0.0265 \cdot X_3^2$	0.3783
**Dispersibility (s)**	$Y = - 210.90 + 6.595 \cdot X_1 - 99.562 \cdot X_2 + 12.525 \cdot X_3 - 0.05 \cdot X_1^2 + 78.90 \cdot X_2^2 - 0.817 \cdot X_3^2$	0.5217
**Wettability (s)**	$Y = - 4809.19 + 127.34 \cdot X_1 + 89.93 \cdot X_2 + 326.53 \cdot X_3 - 1.04 \cdot X_1^2 - 1.64.86 \cdot X_2^2 - 23.91 \cdot X_3^2$	0.6631
**d (0.1) (μm)**	$Y = - 1916.3 - 90.9 \cdot X_1 + 11058.8 \cdot X_2 + 623.5 \cdot X_3 + 0.8 \cdot X_1^2 - 10873.2 \cdot X_2^2 - 50.5 \cdot X_3^2$	0.2339
**d (0.5) (μm)**	$Y = - 446.1 - 124.5 \cdot X_1 + 15887.8 \cdot X_2 + 148.2 \cdot X_3 + 1.1 \cdot X_1^2 - 15776.4 \cdot X_2^2 - 12.9 \cdot X_3^2$	0.2345
**d (0.9) (μm)**	$Y = 4414.9 + 1.7 \cdot X_1 + 17000.8 \cdot X_2 - 769.9 \cdot X_3 + 1.7 \cdot X_1^2 - 16816.0 \cdot X_2^2 + 60.8 \cdot X_3^2$	0.3825
**D [3,2] (μm)**	$Y = - 1092.3 - 118.2 \cdot X_1 + 12670.0 \cdot X_2 + 518.7 \cdot X_3 + 1.0 \cdot X_1^2 - 12304.9 \cdot X_2^2 - 42.8 \cdot X_3^2$	0.2451
**span**	$Y = - 0.2113 + 0.4391 \cdot X_1 - 33.0087 \cdot X_2 - 1.0347 \cdot X_3 - 0.004 \cdot X_1^2 + 32.4870 \cdot X_2^2 + 0.088 \cdot X_3^2$	0.3392
