# Supplementary material for: Development of Novel Honey- and Oat-Based Cocoa Beverages—A Comprehensive Analysis of the Impact of Drying Temperature and Mixture Composition on Physical, Chemical and Sensory Properties
Source: Molecules. 2024 Sep 30;29(19):4665. doi: 10.3390/molecules29194665 (PMC11477636; doi:10.3390/molecules29194665)
Supplement: Supplementary file 1 [file molecules-29-04665-s001.zip › Supplementary Table S2.pdf]

Supplementary Table S2. RSM models for description of chemical properties of extracts (X_1 temperature, X_2 honey oat flour ratio, X_3 proportion of cocoa powder)

	Model equation	R^2
**TDS (mg L^{-1})**	$Y = 94.967 – 0.512 \cdot X_1 + 161.926 \cdot X_2 – 23.464 \cdot X_3 + 0.009 \cdot X_1^2 – 214.444 \cdot X_2^2 + 2.084 \cdot X_3^2$	0.5726
**Conductivity (μS cm^{-1})**	$Y = 189.128 – 0.94 \cdot X_1 + 331.463 \cdot X_2 – 48.015 \cdot X_3 + 0.017 \cdot X_1^2 – 436.852 \cdot X_2^2 + 4.255 \cdot X_3^2$	0.5712
**pH**	$Y = 14.6585 – 0.3459 \cdot X_1 – 1.0463 \cdot X_2 + 0.3998 \cdot X_3 + 0.003 \cdot X_1^2 + 1.3888 \cdot X_2^2 – 0.028 \cdot X_3^2$	0.9248
**Brix (°)**	$Y = - 8.3271 + 0.6495 \cdot X_1 + 6.1574 \cdot X_2 + 1.8 \cdot X_3 – 0.005 \cdot X_1^2 – 6.0185 \cdot X_2^2 – 0.1363 \cdot X_3^2$	0.5815
**L***	$Y = - 76..113 + 5.259 \cdot X_1 – 311.432 \cdot X_2 + 9.320 \cdot X_3 – 0.042 \cdot X_1^2 + 314.587 \cdot X_2^2 – 0.753 \cdot X_3^2$	0.6334
**a***	$Y = 2.0622 – 0.263 \cdot X_1 – 32.4352 \cdot X_2 – 0.3098 \cdot X_3 + 0.002 \cdot X_1^2 – 32.8333 \cdot X_2^2 + 0.0295 \cdot X_3^2$	0.4025
**b***	$Y = - 14.9185 – 0.0023 \cdot X_1 + 44.8537 \cdot X_2 + 1.032 \cdot X_3 – 0.0023 \cdot X_1^2 – 44.7222 \cdot X_2^2 – 0.0768 \cdot X_3^2$	0.5112
**Chroma**	$Y = - 14.3036 + 0.1697 \cdot X_1 + 50.0.611 \cdot X_2 – 0.9659 \cdot X_3 – 0.0018 \cdot X_1^2 – 49.9815 \cdot X_2^2 – 0.0706 \cdot X_3^2$	0.4919
**Hue**	$Y = - 3.612 – 0.2302 \cdot X_1 – 10.601 \cdot X_2 – 0.2868 \cdot X_3 – 0.2302 \cdot X_1^2 + 10.9134 \cdot X_2^2 + 0.0442 \cdot X_3^2$	0.4088
**TPC (mg GAE g_{dm} $^{-1}$)**	$Y = 13.0857 + 5.259 \cdot X_1 – 311.432 \cdot X_2 + 9.320 \cdot X_3 – 0.042 \cdot X_1^2 + 314.587 \cdot X_2^2 – 0.753 \cdot X_3^2$	0.4678
**DPPH (mmol TE g_{dm} $^{-1}$)**	$Y = -0.01419 – 0.00215 \cdot X_1 – 0.01206 \cdot X_2 – 0.01389 \cdot X_3 – 0.000014 \cdot X_1^2 + 0.010717 \cdot X_2^2 + 0.001191 \cdot X_3^2$	0.6692
**FRAP (mmol $FeSO_4$ g_{dm} $^{-1}$)**	$Y = - 0.00889 – 0.0004 \cdot X_1 + 0.09347 \cdot X_2 + 0.00182 \cdot X_3 + 0.000007 \cdot X_1^2 – 0.09515 \cdot X_2^2 + 0.000146 \cdot X_3^2$	0.6597
