# Supplementary material for: Development of Novel Honey- and Oat-Based Cocoa Beverages—A Comprehensive Analysis of the Impact of Drying Temperature and Mixture Composition on Physical, Chemical and Sensory Properties
Source: Molecules. 2024 Sep 30;29(19):4665. doi: 10.3390/molecules29194665 (PMC11477636; doi:10.3390/molecules29194665)
Supplement: Supplementary file 1 [file molecules-29-04665-s001.zip › Supplementary Table S3.pdf]

Supplementary Table S3. RSM models for description of sensory properties of powders and beverages (X_1 temperature, X_2 honey oat flour ratio, X_3 proportion of cocoa powder)

Ouput variable		Model equation	R^2
powder	appearance	$Y = 9.1777 – 0.0344 \cdot X_1 + 6.2222 \cdot X_2 – 1.84 \cdot X_3 + 0.0003 \cdot X_1^2 – 6.6667 \cdot X_2^2 + 0.1493 \cdot X_3^2$	0.1667
	color	$Y = 8.1185 – 0.1656 \cdot X_1 + 12.7778 \cdot X_2 – 0.8356 \cdot X_3 + 0.0014 \cdot X_1^2 – 12.222 \cdot X_2^2 + 0.0711 \cdot X_3^2$	0.3502
	odour	$Y = 1.8889 + 0.07 \cdot X_1 – 1.8889 \cdot X_2 - 0.24 \cdot X_3 - 0.0003 \cdot X_1^2 + 3.3333 \cdot X_2^2 + 0.02133 \cdot X_3^2$	0.5414
bevarage	appearance	$Y = 5.3407 + 0.1756 \cdot X_1 - 14.778 \cdot X_2 – 0.8711 \cdot X_3 – 0.0016 \cdot X_1^2 + 14.444 \cdot X_2^2 + 0.0711 \cdot X_3^2$	0.3654
	color	$Y = 3.3926 + 0.5111 \cdot X_1 – 2.66667 \cdot X_2 – 0.1067 \cdot X_3 - 0.0004 \cdot X_1^2 + 2.2222 \cdot X_2^2 + 0.0142 \cdot X_3^2$	0.1444
	odour	$Y = - 13.9778 + 0.4156 \cdot X_1 + 13.3333 \cdot X_2 + 0.5511 \cdot X_3 – 0.0033 \cdot X_1^2 – 13.3333 \cdot X_2^2 – 0.0427 \cdot X_3^2$	0.5303
	sweetness	$Y = - 5.3734 + 0.02 \cdot X_1 + 6.7778 \cdot X_2 + 2.0533 \cdot X_3 – 0.0002 \cdot X_1^2 – 0.5.5556 \cdot X_2^2 – 0.1635 \cdot X_3^2$	0.2773
	bitterness	$Y = 17.9037 – 0.8111 \cdot X_1 + 8.5556 \cdot X_2 + 2.17778 \cdot X_3 + 0.0069 \cdot X_1^2 – 7.7778 \cdot X_2^2 – 0.1778 \cdot X_3^2$	0.6594
	taste	$Y = 3.6889 – 0.2289 \cdot X_1 + 4.7778 \cdot X_2 + 1.5822 \cdot X_3 + 0.002 \cdot X_1^2 – 3.3333 \cdot X_2^2 – 0.128 \cdot X_3^2$	0.3306
	texture	$Y = 1.2667 + 0.1922 \cdot X_1 – 9.6667 \cdot X_2 – 0.2578 \cdot X_3 - 0.0017 \cdot X_1^2 + 10.00 \cdot X_2^2 + 0.0213 \cdot X_3^2$	0.1981
